# Prevalence of Cardioprotective Medication Use in Coronary Heart Disease Patients in South America: Systematic review and Meta-Analysis

**DOI:** 10.5334/gh.1124

**Published:** 2022-06-08

**Authors:** A. Marzà-Florensa, E. Drotos, P. Gulayin, D. E. Grobbee, V. Irazola, K. Klipstein-Grobusch, I. Vaartjes

**Affiliations:** 1 Julius Global Health, Julius Center for Health Sciences and Primary Care, University Medical Center Utrecht, Utrecht University, Utrecht, The Netherlands; 2 Department of Health Promotion, Care and Public Health Research Institute, Maastricht University, Maastricht, The Netherlands; 3 Instituto de Efectividad Clínica y Sanitaria, Buenos Aires, Argentina

## Abstract

**Background:**

Coronary heart disease (CHD) is the most common cause of death globally, and clinical guidelines recommend cardioprotective medications for patients with established CHD. Suboptimal use of these medications has been reported, but information from South America is scarce.

**Methods:**

We conducted a systematic review on prevalence of secondary prevention medication in South America. We pooled prevalence estimates, analysed time-trends and guideline compliance, and identified factors associated with medication use with meta-regression models.

**Results:**

73 publications were included. Medication prevalence varied by class: beta-blockers 73.4%(95%CI 66.8%–79.1%), ACEI/ARBs 55.8%(95%CI 49.7%–61.8), antiplatelets 84.6%(95%CI 79.6%–88.5%), aspirin 85.1%(95%CI 79.7%–89.3%) and statins 78.9%(95%CI 71.2%–84.9%). The use of beta-blockers, ACEI/ARBs and statins increased since 1993. Ten publications reported low medication use and nine reported adequate use. Medication use was lower in community, public and rehabilitation settings compared to tertiary centres.

**Conclusion:**

Cardioprotective medication use has increased, but could be further improved particularly in community settings.

## Introduction

Coronary heart disease (CHD) is the main cause of death and one of the most important causes of disability worldwide and in South America [1]. Cardioprotective medications, including antiplatelet, anti-hypertensive, lipid-lowering and hypoglycaemic medication, are effective in preventing CHD morbidity and mortality, [2,3,4] and their long-term use in patients with established CHD is recommended by international guidelines [2,5].

Despite guideline recommendations, research shows that the use of these medications in secondary prevention of CHD patients is suboptimal [3,6,7]. This gap between guideline recommendations and clinical use has been described in high-income countries [4,8,9,10,11], but information from middle-and and low income countries, including the South American region [12], is limited. Meta-analyses have been conducted to explore this problem in North America, Europe [9], and China [13], and there is high variability by region [14] in the use of guideline-recommended medications for CHD secondary prevention. To date, an overview and general picture of secondary prevention medication and its determinants in South America is lacking. Therefore, the aim of this systematic review is to summarize evidence on the prevalence of cardioprotective medication use for secondary prevention of CHD in South America. The secondary aims of this work are to summarize the findings on guideline compliance, examine time trends and identify potential factors associated with use of medication in patients with established CHD.

## Methods

### Search strategy

This review was registered with PROSPERO (registration number CRD42020206657) and conducted in accordance with the PRISMA guidelines [15] (Supplementary File 1). We conducted a systematic search on April 28^th^, 2021 on the following databases: PubMed, Embase, Cochrane, LILACS and SciELO. The search strategy contained information on the CHD diagnosis of the patients, the country where the study was performed and the most common classes of cardioprotective medications in the outpatient clinic setting. Studies published between 2000 and 2021 in English, Spanish or Portuguese and conducted in South America (Argentina, Bolivia, Brazil, Chile, Colombia, Ecuador, Guyana, Paraguay, Peru, Suriname, Uruguay and Venezuela) reporting the prevalence of cardioprotective medications in CHD patients in outpatient settings were included. Broad terms were included for the diagnosis of CHD: ‘coronary artery disease,’ ‘myocardial infarction,’ ‘ST-elevation myocardial infarction,’ ‘non-ST-elevation myocardial infarction,’ ‘acute coronary syndrome,’ ‘angina pectoris,’ ‘acute coronary syndrome,’ ‘coronary atherosclerosis,’ interventions such as ‘coronary artery bypass graft’ and ‘percutaneous coronary intervention,’ and commonly used acronyms for these terms. For details on the search strategy and PROSPERO registration see Supplementary File 2.

### Eligibility criteria

The classes of cardioprotective medications taken into account were anti-platelet drugs, lipid-lowering drugs, antihypertensive agents (beta-blockers, ACE-inhibitors, ARBs, diuretics, and nitrates), oral hypoglycaemics and insulin. Intervention studies (randomized clinical trials and non-randomized interventions) and observational studies (cross-sectional, cohort and case-control studies) were included. Case reports, case series, reviews, as well as publication types other than original articles were excluded.

### Study selection

The publications resulting from the search were screened by the above eligibility criteria on their titles and abstracts using the platform Rayyan CQRI [16]. Screening was conducted by two reviewers (ED, AMF). Each reviewer screened half of the articles, and an additional 10% of the articles was screened by the other reviewer to prevent interpersonal bias. The reviewers discussed discrepancies and unclear decisions until consensus was reached. The publications that fulfilled the inclusion and exclusion criteria were screened on their full-text following the same strategy.

### Data extraction

Relevant data was extracted from the selected publications. Data extraction was performed using the electronic data capture system REDCap©[17] by two reviewers (ED, AMF). Each reviewer extracted data from the articles that the other reviewer had previously screened to minimize potential bias. Collected data included information on authors, publication year, publication title, name of the study, language, period in which the study was conducted, country, study design; participants characteristics including specific diagnosis like CHD, acute coronary syndrome (ACS), coronary artery bypass graft (CABG) or percutaneous coronary intervention (PCI); percentage of women, age range, mean age, socioeconomic status (including percentage of participants in the highest income and education categories as well as percentage of employment), and cardiovascular risk factors (blood pressure, body mass index, lipids and glucose levels), care setting information (type of hospital or healthcare centre, e.g. primary care, academic hospital, tertiary hospital, rehabilitation, and whether the centre was public or private), and urbanicity.

Outcome data included the prevalence of medication per medication class. In the case of drug intervention studies, we extracted data on medication prevalence at baseline. In publications with an observational design that reported medication prevalence at multiple time-points, we extracted data from the earliest time-point in order to facilitate comparison with intervention studies. If not reported directly, medication prevalence was calculated when possible.

Secondary outcome data included guideline compliance (report of compliance or non-compliance), time trends (starting year of the study) and determinants associated with use of medication in patients with established CHD (outcomes reported in stratified analysis or coefficients reported in regression models).

### Quality assessment

A tool for the quality assessment of studies reporting prevalence estimates was adapted from the previous work by Zhao et al. [13], and Li et al. [18] (Supplementary File 3A). For overall risk of bias assessment, we summarized risk of bias as follows: for risk of bias in each domain (study design, study population, participation rate, participants’ characteristics and outcome) 2 points were given for low risk, 1 point for moderate risk and 0 points for high or unclear risk. Publications with a score lower than 6 out of 10 were excluded. The remaining publications were classified as: moderately low risk of bias (6–7 points), low risk of bias (8–9 points) and very low risk of bias (10 points). Reviewers ED and AMF assessed the quality of articles for which they extracted the data, and additionally they assessed the quality of 10% of the articles examined by the other reviewer. Discrepancies were discussed until consensus was achieved.

### Data analysis

Data analysis was conducted using R Studio [19]. Data on medication prevalence is expressed in percentages by class of medication. We reported the prevalence of each kind of medication separately. We presented separate categories for those articles reporting general classes of medications instead of specific drugs. In the case of antiplatelet drugs, we additionally showed the estimates of articles reporting the prevalence of aspirin, clopidogrel, and not-specified antiplatelet drugs combined because of the shared indication for these medications, and to be able to explore the use of this medication class in general.

Meta-analysis was performed using a mixed model from the R package ‘metafor’ for each class of medication [20]. The results were expressed as pooled prevalence with 95% confidence intervals (CI) and random effects, and displayed in forest plots by care setting. Heterogeneity was quantified with the I^2^ test. The same statistical package was used in a sensitivity analysis to analyse potential differences in prevalence between studies conducted in Brazil and in other countries.

In order to explore time trends in medication use, mixed meta-regression models were fitted with the starting year of the study as covariate for each medication class. The reported prevalence of medication and the model prediction were plotted against the year the studies commenced in bubble plots to illustrate time-trends in medication use.

Meta-regression models were performed to discern potential factors contributing to medication use. A mixed meta-regression model was run for each class of medication, including the following covariates: the proportion of women included, time of outcome measurement since the start of the study, diagnosis of the patients included in the study, urban region, and care setting. The full models were reduced and simpler models were compared against the full models and among them with the AIC fitting statistic. Models with the lowest AIC were selected. Results were expressed as odds ratios (OR) and 95% CI.

## Results

### Study selection

**Figure 1:**
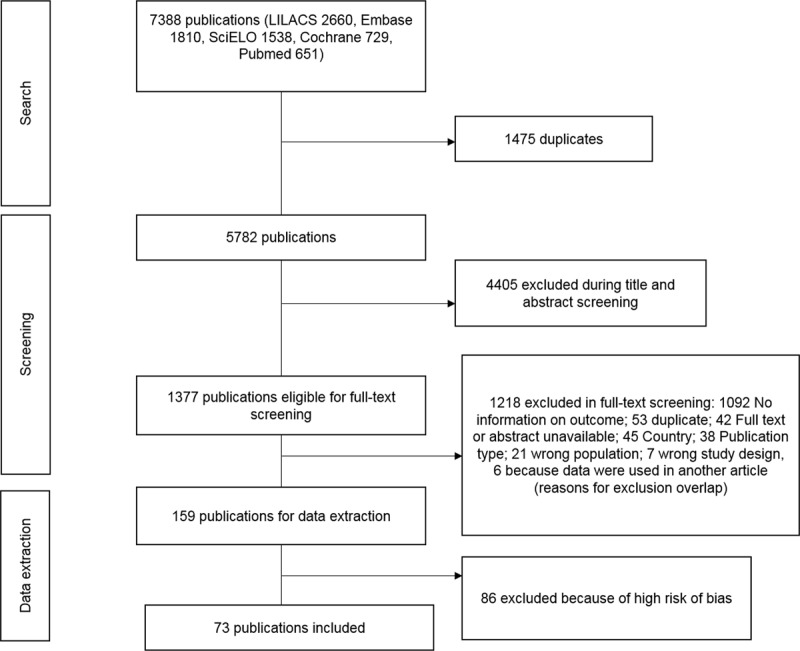
Study selection flow-chart.

The search strategy resulted in 7388 publications: 2660 in LILACS, 1810 in Embase, 1538 in SciELO, 729 in Cochrane and 651 in PubMed. After removing 1606 duplicates, 5782 publications were screened on their title and abstract. 4405 publications did not fulfil the inclusion criteria and were excluded, resulting in 1377 publications eligible for full-text screening. During a full-text screening, 1218 articles were excluded (Figure 1). Of the remaining 159 publications, 86 did not reach the quality threshold during the quality assessment, and therefore 73 publications were finally included in the review.

### Study characteristics

Table 1 describes the main characteristics of the included studies. All articles included were published between 2000 and 2020, referring to studies conducted between 1993 and 2017. Most studies were conducted in Brazil [3,21,22,23,24,25,26,27,28,29,30,31,32,33,34,35,36,37,38,39,40,41,42,43,44,45,46,47,48,49,50,51,52,53,54,55,56,57,58]. Six studies were conducted in Argentina [59,60,61,62,63,64], four in Chile [65,66,67,68], four in Colombia [69,70,71,72], three in Uruguay [73,74,75] and two were multi-country studies conducted in Argentina, Brazil, Chile and Colombia [12] and Brazil and Suriname [76]. The most common language of the articles was English (58 articles), followed by Spanish (11 articles) and Portuguese (4 articles).

**Table 1:** Characteristics of the studies included in the review.


PUBLICATION	STUDY DURATION	COUNTRY	STUDY DESIGN	N	CARE SETTING	DIAGNOSIS CATEGORY	URBAN SETTING	% WOMEN	AGE	SOCIOECONOMIC STATUS

Castillo y Costa, 2018	NA-2015	Argentina	Cohort	210		MI, CABG, PCI	Unclear	17.0	59.0 (9); 61.0 (9.0)	

Fernandes, 2012	2003–2004	Brazil	RCT	45		PCI		38.0	62.7 (9.9), 26–89	

Gurfinkel, 2004	2001-NA	Argentina	RCT	301		ACS	Urban		59 (8.7), 59 (7.9)	

Ladeia, 2003	1995–1997	Brazil	Cross-sectional	104		CHD	Urban	32.7	60.9 (8.1)	Education: 10.6

Lima-Filho, 2010	2001–2002	Brazil	Cohort	70		PCI		22.9	57.6 (13.9), 59.4 (7.6)	

Lorenzo, 2014	2008–2010	Brazil	Cohort	228		CHD	Urban	46.1	63.15 (12.26)	

Baptista, 2012	2009–2011	Brazil	Cohort	97	Academic or Tertiary Hospital	CABG		33.3	63.5 (9.4), 42–81	

Bohatch, 2015	2011–2013	Brazil	Cohort	230	Academic or Tertiary Hospital	CABG		24.3		

Brasil, 2013	NA-NA	Brazil	Cross-sectional	710	Academic or Tertiary Hospital	CHD	Urban		57.4 (4.1)	

Breda, 2008	2008–2005	Brazil	RCT	50	Academic or Tertiary Hospital	CABG	Urban	42.0	62.1 (12)	

Chaves, 2004	2001–2002	Brazil	RCT	96	Academic or Tertiary Hospital	CHD	Urban	51.0	65.07 (12.49)	

Chaves, 2019	2015–2017	Brazil	RCT	115	Academic or Tertiary Hospital	CABG, PCI	Urban	28.7	63.9 (10. 9), 63 (12.1)	Employmnent: 40

Cruz, 2009	2004–2005	Brazil	Cross-sectional	103	Academic or Tertiary Hospital	CHD			67.9 (12.3)	

Dayan, 2018	2006–2014	Uruguay	retrospective	282	Academic or Tertiary Hospital	CABG		26.6	65.58 (9.5), 61.75 (9.6)	

Feguri, 2017	2014–2016	Brazil	RCT	574	Academic or Tertiary Hospital	CABG	Urban	33.0	62.12 (9.63), 60.93 (8.91)	

Fernandez, 2011	2006–2007	Colombia	RCT	400	Academic or Tertiary Hospital	PCI	Urban	45.0	58.0 (9.0)	

Furuya, 2014	2011–2012	Brazil	RCT	60	Academic or Tertiary Hospital	PCI	Urban	43.0	56.9 (10.8), 34–85	Employment: 35.0

Gomes, 2011	2002–2006	Brazil	Cohort	504	Academic or Tertiary Hospital	PCI	Urban	35.9	63.7 (11.0)	

Hueb, 2004	1995–2000	Brazil	RCT	611	Academic or Tertiary Hospital	CHD	Urban	15.0	60.25 (9.26), 58.92 (6.04)	

Kimura, 2018	2007–2013	Brazil	Cohort	520	Academic or Tertiary Hospital	CABG	Urban	72.1		

Liberato, 2016	2010–2011	Brazil	Cross-sectional	190	Academic or Tertiary Hospital	ACS	Urban	36.1	64.9, 32–93	Employment: 31.0

Nazzal, 2013	2008–2008	Chile	Registry	416	Academic or Tertiary Hospital	ACS	Urban	23.4		Income: 20.0

Neira, 2013	2011–2011	Chile	Cross-sectional	202	Academic or Tertiary Hospital	CHD	Urban	29.7	58.9 (9.8), 60.6 (8.5)	Education: 17.4 Employment: 45.0

Nery, 2015	2009–2012	Brazil	RCT	61	Academic or Tertiary Hospital	ACS	Urban	27.9	59.5 (9.4)	

Neves, 2012	NA-NA	Brazil	descriptive, cross-sectional study	20	Academic or Tertiary Hospital	CHD		0.0		

Noriega, 2008	NA-NA	Chile	Non-randomized intervention	64	Academic or Tertiary Hospital	CABG, PCI		20.3	64.0 (11.0), 63 (12.0)	

Oliveira, 2019	2013–2015	Brazil	Retrospective cohort	536	Academic or Tertiary Hospital	ACS	Urban	36.0	65.6	Education: 49.2; Income: 34.0

Pantoni, 2016	NA-NA	Brazil	Non-randomized intervention	27	Academic or Tertiary Hospital	CABG	Urban	44.4	60.0 95% CI 51–68), 63.0 (95% CI 55–70), 61.0 (95% CI 53–73)	

Pellegrini, 2014	2002–2007	Brazil	Cohort	611	Academic or Tertiary Hospital	ACS	Rural	28.6	61.4 (11.6)	

Pesaro, 2012	2006–2009	Brazil	RCT	78	Academic or Tertiary Hospital	CHD	Urban	38.5	64.0 (12.0), 65.0 (12.0), 61.0 (12.0)	

Portal, 2003	1998–1999	Brazil	RCT	39	Academic or Tertiary Hospital	CHD		43.6	62,7 (10.7), 61.6 (11.1)	

Ribeiro, 2015	2007–2008	Brazil	Cross-sectional	153	Academic or Tertiary Hospital	PCI	Urban	49.0	61.9 (11.9)	

Ribeiro, 2018	2014–2016	Brazil	Cohort	169	Academic or Tertiary Hospital		Urban	16.0	63.7 (9.6)	

Rossi, 2014	2006–2006	Argentina	Cohort	125	Academic or Tertiary Hospital	ACS	Urban	34.4	56.0 (9.0), 60.0 (9.0)	

Rueda-Clausen, 2010	2005–2006	Colombia	Cross-sectional	34	Academic or Tertiary Hospital	CHD	Urban	23.5	64.0, 61.0	

Saffi, 2013	2008–2010	Brazil	RCT	74	Academic or Tertiary Hospital	CHD		26.0	60.9(10.6), 63.4 (8.56), 59.9(11.8), 62.7(10.9)	Income: 58.0

Santos, 2015	2007–2010	Brazil	Cohort	198	Academic or Tertiary Hospital	PCI		30.3	55.0 (8.0), 52.0 (7.0), 54.0 (10.0)	

Scherr, 2010	1997–2002	Brazil	Non-randomized intervention	2337	Academic or Tertiary Hospital	CHD	Urban	39.2	64.3 (10.7), 64.5 (10.9)	

Silva, 2005	1995–1998	Brazil	RCT	210	Academic or Tertiary Hospital	CHD	Urban	32.4	60.2 (10), 28–87	

Silveira, 2007	2002–2003	Brazil	RCT	24	Academic or Tertiary Hospital	CABG		37.5	58.5 (9.4)	

Silveira, 2008	1998–2005	Brazil	Cohort	310	Academic or Tertiary Hospital	CHD	Unclear	39.0		

Simon, 2019	2014–2015	Brazil	RCT	48	Academic or Tertiary Hospital	ACS		35.4		

Siniawski, 2019	2014–2017	Argentina	Cross-sectional	351	Academic or Tertiary Hospital	ACS, CABG	Urban	26.5	63.3 (12.4), 60.0 (87)	

Smidt, 2009	2002–2007	Brazil	Registry	611	Academic or Tertiary Hospital	ACS		36.6	60.9 (10.3), 31–81	

Souza Groia Veloso, 2020	NA-NA	Brazil, Suriname	Cross-sectional	148	Academic or Tertiary Hospital	CHD	Unclear	29.7	Median 61.0 (IQR 54–68)	

Souza, 2013	2008–2010	Brazil	Registry	103	Academic or Tertiary Hospital	ACS	Urban	16.5	62.6 (9.3), 63.3 (11.3)	

Uchoa, 2015	NA-NA	Brazil	Cohort	67	Academic or Tertiary Hospital	CHD, CABG	Urban	25.0	61.2 (10.0), 68.6 (9.0)	

Vilar, 2015	2009–2010	Brazil	Cross-sectional	155	Academic or Tertiary Hospital	CHD		18.7	60.0 (9.0)	

Villacorta, 2012	2006–2008	Brazil	Cohort	209	Academic or Tertiary Hospital	PCI	Urban	26	Median 62.0 [IQR 17.0]	

Abreu-Silva, 2011	2008–2010	Brazil	Registry	535	Other	PCI		32.0	67.0 (10.4)	

Alvarez, 2016	1993–2013	Argentina	Cross-sectional	866	Other	ACS		24.0	62.7 (11.1)	

Berwanger, 2013	NA-NA	Brazil	Cross-sectional	681	Other	ACS				

Fernandez, 2009	2003–2006	Colombia	Cohort	395	Other	CHD		32.7	64.4 (12.9), 66.8 (10.9)	

Finimundi, 2007	NA-NA	Brazil	RCT	40	Other	ACS	Urban	43.0	60.1 (2.2), 63.21 (2.21)	

Gaedke, 2015	NA-NA	Brazil	Cohort	138	Other	ACS	Urban	44.4	62.5 (11.1)	Education: 54.8 Income: 33.3

Gowdak, 2007	1998–2004	Brazil	Cohort	119	Other	CHD	Urban		57.4 (5.9), 58.3 (8.6)	

Mattos, 2012	2010–2011	Brazil	Registry	2475	Other	ACS		32.2	64 (8.0), 65 (9.0), 66 (8.0)	

Mendis, 2005	2002–2003	Brazil	Cross-sectional	836	Other	CHD	Both		56.0 (10.0)	

Vazquez, 2011	2008–2009	Uruguay	Cohort	154	Other	ACS		21.4		

Vesga, 2006	NA-NA	Colombia	Cross-sectional	71	Other	CHD	Urban	28.2	58.4 (7.9)	

Avezum, 2017	2003–2009	Argentina, Brazil, Chile, Colombia	Cross-sectional	910	Primary Care/Community	CHD	Urban and rural	61.3	62.20 (11.60)	

Vianna, 2012	2008–2008	Brazil	Cross-sectional	295	Primary Care/Community	ACS	Urban			

Birck, 2019	2008–2010	Brazil	Cross-sectional	405	Primary Care/Community	CHD	Urban	36.5	61.6 (9.4)	Education: 48.6, Income: 38.3

Stockins, 2011	2005–2006	Chile	Cohort	233	Publi Hospital	ACS		30.6	68.0	

Aguiar, 2010	1999–2007	Brazil	Cohort	377	Public Hospital	ACS		37.9	62.3 (9.3)	

Carvalho, 2007	1992–2000	Brazil	Retrospective cohort	381	Rehabilitation			19.4		

Gambogi, 2009	2004–2006	Uruguay	Cohort	900	Rehabilitation		Both	25.3	57.9 (9.9), 61.3 (7.7)	Education: 9.5 Employment: 44.6

Garlet, 2017	2015–2016	Brazil	Cross-sectional	102	Rehabilitation	CHD		31.4	61.7 (10.0), 64.5 (9.0)	

Lelys, 2019	2015–2017	Brazil	Cross-sectional	115	Rehabilitation	CHD		28.7	59.9(8.6); 57.2 (9.0)	Employment: 40.0

Pantoni, 2014	2006–2008	Brazil	Non-randomized intervention	28	Rehabilitation	CABG	Urban	32.1	56.0	

Fuchs, 2009	2005–2006	Brazil	Cross-sectional	39	Rehbilitation	CHD	Urban	10.3	63.7(95% CI 56.6–73.9)	

Castro, 2018	2018–NA	Brazil	Cohort	525	Secondary Hospital	ACS	Urban	39.8	61.6 (11.9)	

Trivi, 2018	2010–2011	Argentina	Cohort	438	Secondary Hospital	ACS		24.2	59.2 (7.9)	


Age is expressed in percentage (standard deviation) unless indicated otherwise; multiple values are given if age was reported by subgroups in the publication. Socioeconomic status indicates the percentage of participants included in the highest category of education or income, or percentage of employed participants. Abbreviations: RCT (randomized controlled trial), ACS (acute coronary syndrome), CABG (coronary artery bypass graft), CHD (coronary heart disease), PCI (percutaneous coronary intervention).

In terms of study design, most publications reported on cohort studies (23 articles), cross-sectional studies (20 publications), and baseline data of randomized clinical trials (17 articles). The number of participants included in each study ranged from 20 to 2475, with a mean of 328 (SD 424). Most studies were conducted in urban areas (39 studies). Regarding the clinical setting, the majority of studies were conducted in academic or tertiary hospitals (42 articles), six in rehabilitation centres, three in primary care or community settings, two in secondary level hospitals, two in public hospitals, and 12 in other settings.

### Participants’ characteristics

The most common diagnosis of the patients included was coronary heart disease (26 articles), followed by ACS (21 articles), PCI (9 articles), CABG (9 articles) and some articles included patients with more than one diagnosis [5]. Most articles included a majority of male participants. The mean percentage of female participants was 32.0% (SD 11.4%).

12 articles provided information on the socioeconomic status (SES) of participants. Educational attainment was reported by seven publications and the proportion of participants with highest educational attainment ranged from of 9.5% to 49.2%. The percentage of employed participants was reported by five articles and ranged from 33.5% to 45.0%; and the proportion of participants in the highest income category (reported in six articles) varied from 20.0% to 58.0%.

Regarding the risk factors of the study populations, 54 articles reported the prevalence of hypertension (range 45.0–96.0%) and 42 articles provided prevalence values for dyslipidaemia (36.0–96.8%). The prevalence of diabetes was reported in 56 articles (range 7.7% to 100%). The prevalence of overweight was reported in 11 of the included studies (range 28.2% to 93.5%), 5 articles reported the prevalence of obesity (range 15.0%–33.7%); and 16 articles included mean or median BMI values, ranging from 26.1 to 29.0 kg/m^2^.

### Quality assessment

**Figure 2:**
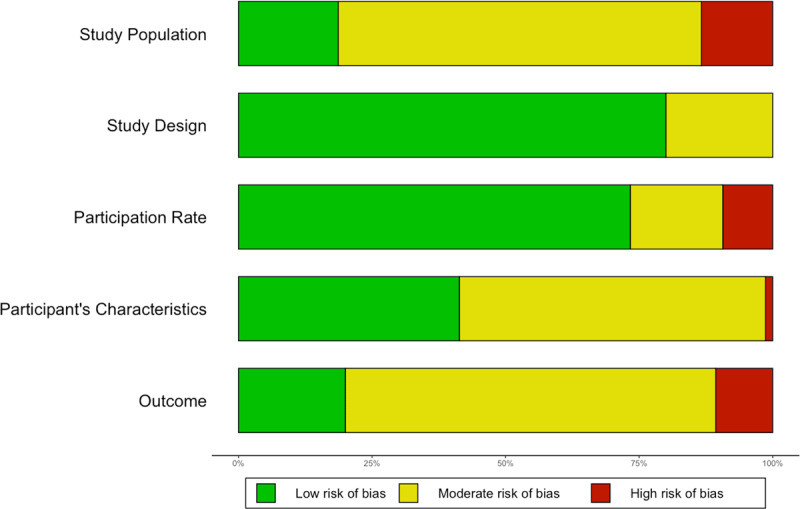
Risk of bias results.

The risk of bias varied by domain of the quality assessment tool: study population was the field in which more articles had a high risk of bias (13.7%), whereas most articles had low risk of bias in the fields of study design (79.5%) and participation rate (72.6%) (Figure 2). The results of the quality assessment of all included publications are displayed in Supplementary Figure 1, and of included and excluded publications in Supplementary File 3B. Supplementary file 3C details the reasons for exclusion of publications with a risk of bias score lower than six.

### Prevalence of medication

**Figure 3:**
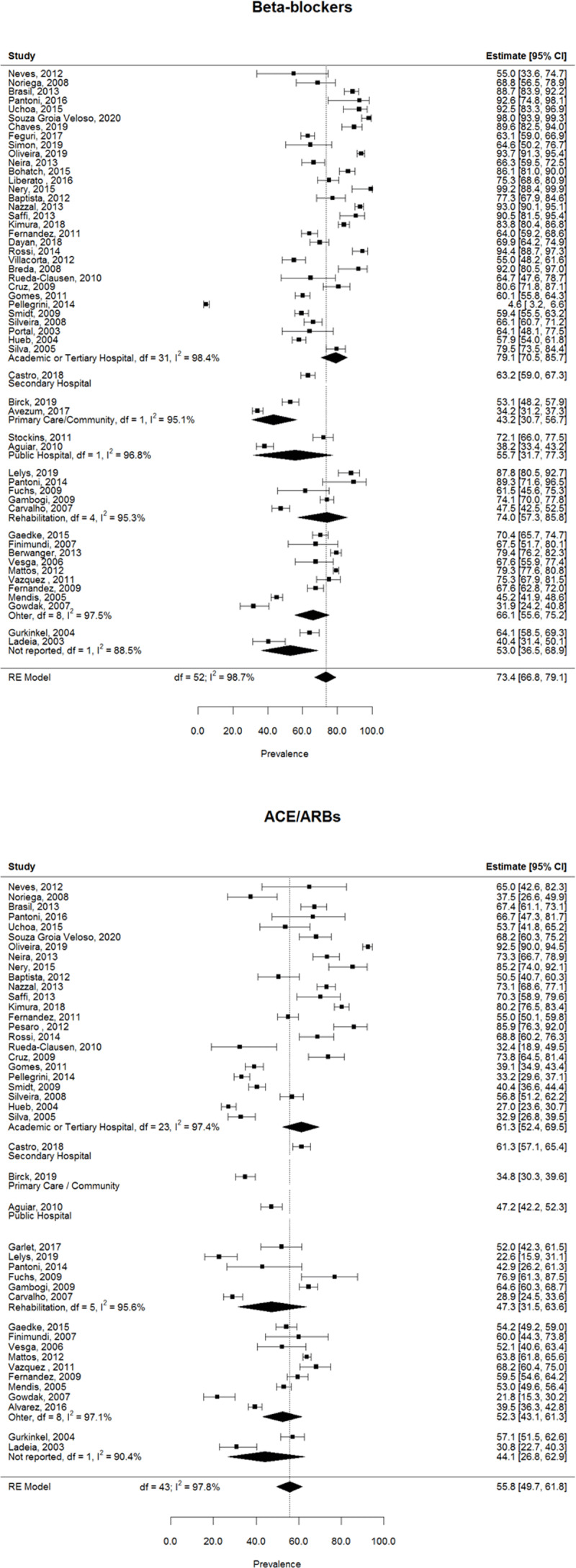
Pooled prevalence of anti-hypertensive medication use.

**Figure 4:**
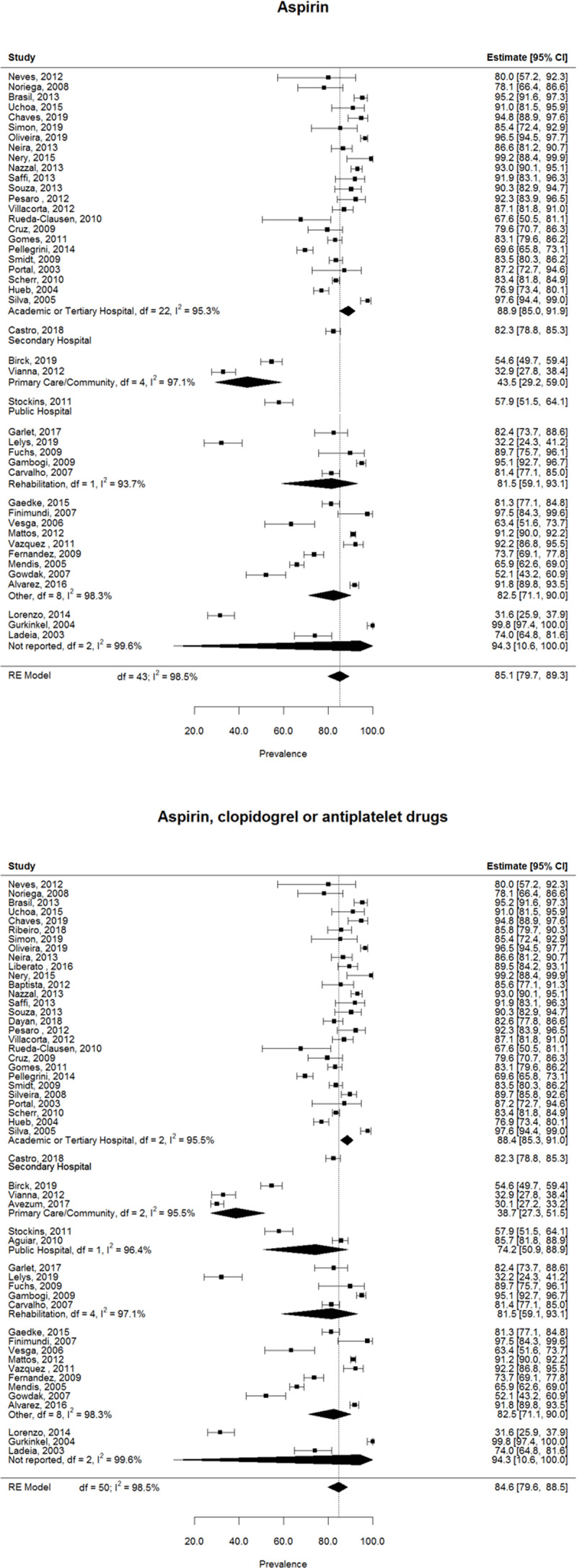
Pooled prevalence of antiplatelet medication use.

**Figure 5:**
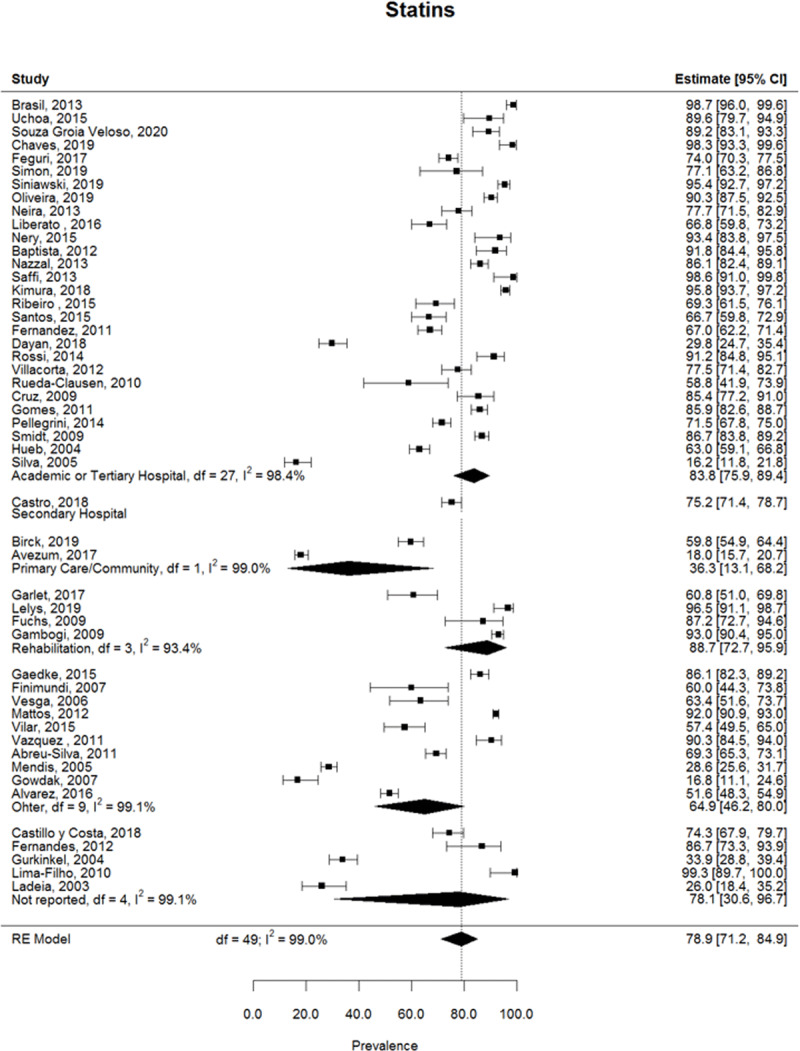
Pooled prevalence of statins.

The prevalence of medication for each study as well as the pooled prevalence estimate per medication is displayed in Figures 3, 4, 5 and summarized in Table 2. The prevalence of beta-blockers was reported in 53 studies, with a pooled estimate of 73.4% (95%CI 66.8% – 79.1%) (Figure 3). 44 articles reported the prevalence of ACEI/ARB use, with a pooled estimate of 55.8% (95%CI 49.7% – 61.8%) (Figure 3). The overall prevalence of antiplatelet drugs (including aspirin, clopidogrel, and articles that didn’t specify the antiplatelet drug) was retrieved from 51 studies, and the pooled prevalence estimate was 84.6% (95%CI 79.6% – 88.5%) (Figure 4). The prevalence of aspirin specifically was retrieved from 44 studies and their pooled estimate was 85.1% (95%CI 79.7% – 89.3%) (Figure 4). The prevalence of statins was reported in 50 articles and the estimated pooled prevalence was 78.9% (95%CI 71.2% – 84.9%) (Figure 5). Total heterogeneity in the meta-analysis models high, ranging from 97.8% (ACEI/ARBs model) to 99.0% (antiplatelet model). No significant differences were observed in the prevalence of any medication classes between Brazil and other countries.

**Table 2:** Summary of the meta-analysis results. Pooled prevalence results are expressed in percentage and 95% confidence interval.


VARIABLE	NUMBER OF STUDIES	POOLED PREVALENCE

**Beta-blockers**	53	73.4 (66.8–79.1)

**ACE inhibitors**	44	55.8 (49.7–61.8)

**Aspirin**	44	85.1 (79.7–89.3)

**Aspirin, clopidogrel or antiplatelet drugs**	51	84.6 (79.6–88.5)

**Statins**	50	78.9 (71.2–84.9)

**Insulin**	9	11.6 (7.0–18.8)

**Antihypertensives (without specification)**	8	46.5 (33.7–59.8)

**Diuretics**	8	30.1 (24.3–36.6)

**Calcium chanel blockers**	6	34.0 (19.4–52.5)

**Nitrates**	6	36.7 (24.1–51.5)

**Antiplatelet (without specification)**	14	75.1 (55.5–87.9)

**Clopidogrel**	13	50.0 (22.9–78.1)

**Dual antiplatelet therapy**	3	80.0 (55.3–92.8)

**Lipid-lowering drugs (without specification)**	2	34.4 (9,1–73.4)

**High-intensity statins**	2	24.1 (6.4-59.8)

**Fibrates**	2	73.1 (69.5–76.5)


The pooled prevalence estimates of medications and medication classes reported by fewer articles is displayed in supplementary Figures 3, 4, 5 and summarized in Table 2. This includes antihypertensive drugs (without specification), diuretics, nitrates, antiplatelet drugs (without specification), calcium channel blockers, clopidogrel, dual antiplatelet therapy, lipid-lowering drugs (without specification), high-intensity statins, and fibrates.

### Time trends

**Figure 6:**
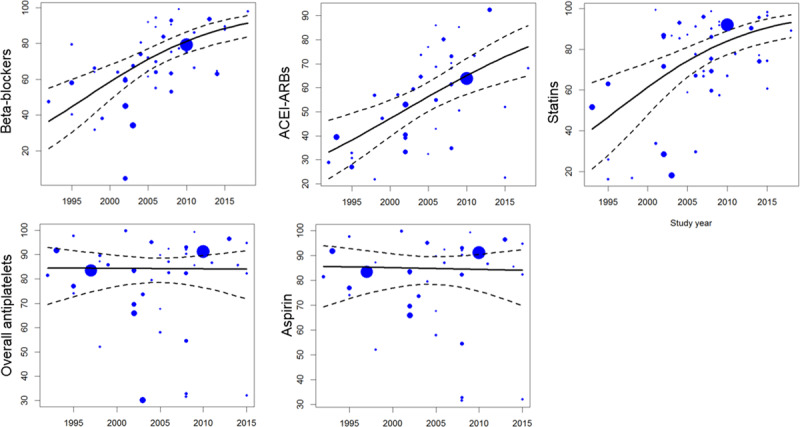
Time trends in medication use. Each circle represents a study and the size of the circle is proportional to the number of participants in the study.

The prevalence of beta-blockers, ACEI/ARBs and statins use significantly increased with time. The use of all antiplatelet drugs and aspirin in particular remained relatively stable over time and the association between use of these medications and year of the study was not significant (Figure 6). The changes in use for other classes of medications were not significant, and they are shown in Supplementary Figure 6. There were too few observations in the lipid-lowering drugs (without specification), high-intensity statins, and fibrates to analyse time-trends.

### Guideline compliance

From the publications included, 19 articles reported whether the prevalence of medication use was adequate. Half of them reported that medication use was low [59,62,76,77,78] or insufficient compared to guideline recommendations [2,12,48,61,79]. Other articles reported that cardioprotective medication use was adequate or high [32,60,66,67,73,75], or in line with guideline recommendations [38,65,80].

### Determinants of medication use

#### Determinants reported in publications

Variables independently associated with medication use included sex, age, socio-economic status, residency, prevalence of cardiovascular risk factors, diagnosis category of CHD patients and health care setting.

It was a common finding that prevalence of cardioprotective medication use was lower in women [2,12,48,77], and younger individuals [12,81]. For example, male patients had an OR ranging from 1.29, (95%CI 1.11–1.49) to 1.54 (95%CI 1.06 – 2.24) for statin use compared to females [12,77]; and patients aged 60 or older presented an OR ranging from 1.42 (95%CI,1.05–1.92) for the use of antiplatelet drugs [12] and 1.94 (1.07–3.50) for the use of cardioprotective medication in general [81].

The presence of cardiovascular risk factors associated with higher medication use. The odds of medication use were higher for overweight (OR of ACEI/ARB use 2.56, 95%CI 1.74–3.77), obese (OR of ACEI/ARB use 2.96, 95%CI 2.00–4.38) and diabetic patients (OR of statin use 1.60, 95%CI 1.08–2.37) [12]. Higher use of aspirin was identified among current [77], and former smokers [81], with OR of 1.83 (95%CI 1.35–2.50) and 1.41 (95%CI 1.03–1.93) respectively compared to non-smokers. High blood pressure was associated with higher use of beta-blockers and ACEI (OR 1.36, 95%CI 1.21–1.52, and 1.74, 95%CI 1.55–1.95 respectively), and high cholesterol with higher use of statins (OR 4.34, 95%CI 3.77–4.99) [77].

A few articles identified the diagnosis category of CHD patients as determinant for medication use. Having a previous PCI was an independent determinant for higher use of antiplatelet drugs (OR 2.00, 95%CI 1.30–2.31) [2], and previous PCI or CABG were associated with higher use of statins (OR 2.37, 95%CI 2.07–2.72) [77]. One publication reported that patients who attended public centres (OR 1.99, 95%CI 1.54–2.59), or centres that are a combination of public and private (1.96, 95%CI 1.51–2.53) had higher odds of cardioprotective medication use compared to those attending private centres [3].

Lower SES [2,12,48,81] and living in rural areas [12] were also associated with lower medication use. In particular, participants from the wealthiest group had an OR of medication use of 2.54 (95%CI 1.08 – 5.95) for use of cardioprotective medication in general, to 5.94 (95%CI 2.80 – 12.6) for statin use compared to the least wealthy group [12,48]; and urban dwellers had an OR of 1.41 (95%CI 1.04–1.92) for use of ACEI/ARB compared to participants from a rural location [12].

#### Meta-regression results

The health care setting, i.e. type of centre where the study had been conducted had a significant effect on medication prevalence for beta-blockers, statins, overall antiplatelet drugs and aspirin: the odds of medication use were lower in studies conducted in primary care and community settings compared to academic and tertiary centres. Further, the odds of overall antiplatelet drugs use were lower in public centres, and the odds of aspirin use were lower in cardiac rehabilitation settings, compared to academic and tertiary centres (Table 3).The use of ACEI/ARBs was not significantly associated with any of the covariates in the meta-regression models. The remaining medications or medication classes presented too few observations and thus meta-regression models could not be fit.

**Table 3:** Results of the meta-regression models showing factors independently associated with medication use. Results are expressed in odds ratios and 95% confidence intervals. Sex was treated as a numerical variable (percentage of women included in the study). The reference category for setting was ‘Academic/Tertiary Hospital,’ and the reference category for diagnosis was ‘coronary heart disease.’ Abbreviations: acute coronary syndrome (ACS), coronary artery bypass graft (CABG), coronary heart disease (CHD), percutaneous coronary intervention (PCI percutaneous coronary intervention). *p = 0.05.


VARIABLE	BETA-BLOCKERS	ACEI ARB	STATIN	ANTIPLATELET DRUGS (OVERALL)	ASPIRIN

**Intercept**	2.56 (0.89–7.37)*	0.84 (0.36–1.94)	3.55 (1.04–12.17)	6.57 (2.60–16.57)*	6.06 (2.18–16.87) *

**Sex**	1.01 (0.98–1.04)	1.01 (0.98–1.04)	1.01 (0.97–1.04)	1.00 (0.97–1.03)	1.00 (0.97–1.03)

**Setting**					

**Primary care/community**	0.18 (0.04–0.96) *		0.11 (0.02–0.62)*	0.12 (0.03–0.40)*	0.19 (0.04–0.96) *

**Public centre**	0.35 (0.07–1.69)			0.28 (0.09–0.86)*	

**Cardiac rehabilitation**	0.92 (0.30–2.78)		1.87 (0.53–6.61)	0.38 (0.14 – 1.04)	0.07 (0.02–0.33) *

**Other**	0.71 (0.27–1.88)		0.67 (0.27–1.64)	0.73 (0.37–1.44)	1.03 (0.36–2.92)

**Diagnosis**					

**ACS**		1.41 (0.78–2.54)		1.65 (0.90–3.03)	1.73 (0.89–3.37)

**PCI**		0.70 (0.21–2.28)		0.92 (0.30 – 2.82)	0.94 (0.29–3.08)

**CABG**		1.21 (0.43–3.37)		0.85 (0.27 – 2.65)	

**CABG, PCI**		0.58 (0.11–2.97)		1.26 (0.38 –4.21)	1.31 (0.37–4.64)


The percentage of women included in the study and the previous CHD diagnosis category of the patient were not significantly associated with the use of any medication class. The number of publications reporting on age, SES and cardiovascular risk factors in a comparable format was low and thus they couldn’t be included in the meta-regression.

## Discussion

### Summary of main findings

The current systematic review shows large variation in the use of cardioprotective medication among CHD patients, ranging from 55.8% for the use of ACEI/ARB drugs to 85.0% for the use of aspirin. A similar number of studies reported suboptimal and adequate guideline compliance. Time-trend analysis for the period 1993 to 2017 showed an increase in the use cardioprotective medication, with the exception of all antiplatelet drugs and aspirin. Use of beta-blockers, statins, overall antiplatelet drugs and aspirin in community settings was lower compared to academic and tertiary centres. The use of antiplatelet drugs in public centres and of aspirin in rehabilitation centres was also lower compared with tertiary centres.

### Prevalence of medication

The prevalence of cardioprotective medication that we observe in South America varies per medication class and shows a general underuse of medications. We observe differences in the prevalence of medication use reported in Europe and North America [8,9,82]. When comparing prevalence estimates found in this review, we observed that the prevalence of antiplatelet drugs and beta-blockers was higher than the estimates found for the PURE study [8] (55.4% of antiplatelet use and 45.4% of beta-blocker use in Europe and Canada) and a systematic review by Naderi et al. (65% of antiplatelet use and 62% of beta-blocker use) [9]. The prevalence of ACEI/ARBs we observed was higher than reported in the PURE Study (46.8% in Europe and North America), but lower than in the review by Naderi et al. (70%) [9]. Prevalence estimates from the international EUROASPIRE IV [82] registry were higher than the ones observed in our review for all medication classes (93.8% for antiplatelets, 82.6% for beta-blockers,58.9% for ACE inhibitors and 27% for insulin) except oral hypoglycaemics (oral sulphonylurea 24.9%) and lipid lowering drugs (fibrates 1.8%). The prevalence of statin use we observed was higher than reported in the PURE study (56.7% in Europe and North America), similar to the prevalence estimate described in a systematic review (76%) [9] and lower than the estimate from EUROASPIRE IV (85%) [82].

However, direct comparison with these studies is challenging because they were conducted in different contexts, regions and time periods and other definitions of medication use. The PURE study was conducted entirely in community settings in high-, low- and middle-income countries and regions. The review by Naderi et al. [9] included studies from high income countries in Europe, North America and Australia, and their definition of medication use was limited to prescription refills. EUROASPIRE IV included a majority of secondary and tertiary level centers and was conducted from 2012–2013, while the present review also included research from community settings and studies that started since 1993 [82].

### Guideline compliance

Most publication included in this review reported that the prevalence of medication use is suboptimal, while others articles find it to be in compliance with guidelines. Despite some publications considering treatment rates high or adequate, we still find that a notable proportion of the patients do not receive guideline-recommended medications: for example, one third of CHD patients were not receiving beta-blockers and almost one fourth were not receiving statins, although these medications are recommended by guidelines. This low use of antihypertensives may be explained by individuals having adequate blood pressure levels or contraindications, despite guidelines recommending these drugs for all CHD patients. Statins, however, are generally well-tolerated drugs and they are recommended to all CHD patients regardless of cholesterol levels. Therefore, the fact that a substantial proportion of patients does not use them may respond to factors other than possible contraindications. Challenges to adhere to guidelines identified by clinicians include difficulties to change usual practice, time pressure and case complexity among others [83].

Articles considering the prescription rates adequate still noted that medication use decreased with time after diagnosis [59,75], indicating that there is room to improve medication adherence and secondary prevention of CHD [59]. It is noteworthy that some publications report that achievement of cardiovascular risk factor targets was inadequate despite high levels of medication use [59,66,67,75,80], which may be attributed to the use of suboptimal doses [59].

### Time trends

We observed increased use of most cardioprotective medications. These trends are in line with large surveys conducted in Europe that report an increase of the use of cardioprotective medications from 1999 and 2004 to 2013 [84,85]. These changes may be attributed in part to the implementation of evidence-based clinical guidelines and public health policies in many South American countries. Mendis et al. [77] previously highlighted the lack of clinical guidelines as a potential factor contributing to the low treatment rates in the PREMISE study. Guideline recommendations have changed in the last decades. For example, the target LDL cholesterol level recommended in guidelines by scientific societies has become lower, from 100 mg/dl in guidelines from 2008 and 2001 [86,87], to 75mg/dl [88], and finally to 70mg/dl in the most recent guidelines [89,90,91,92]. The recommendation to prescribe statins to CHD patients changed accordingly, and the most recent recommendations from scientific societies and clinical guidelines recommend statin use in CHD patients regardless of their cholesterol level. These changes may promote a higher intake of statin use, which is in line with research showing a decrease in cholesterol levels globally and also in the South American region [93].

The gradual investment and unfolding of public healthcare systems with wide coverage, such as the Sistema Unico de Saúde (SUS) in Brazil, has promoted the use of medication by a growing primary care network and provision of drugs free of charge [48]. Although there are still barriers to medication access, the growing coverage of public healthcare systems has allowed more CHD patients to access recommended medications.

### Determinants of medication use

#### Participant’s characteristics

Several studies found female sex and younger age to be independent predictors for lower use of medication [48,94,95,96,97]. Women often receive less prescriptions and have lower adherence to medication, which has been attributed to physician and patients factors like presentation of distinct symptoms, and underestimation of disease severity and fear of side effects [48,94,95,96,97]. In our meta-regression models, however, the percentage of women included in the study was not significantly associated with higher use of any medication class. The lower use of medication in younger patients may be explained by better medication adherence in patients with known risk factors, like older age [12].

High SES showed an independent and strong association with medication use in many studies. Lack of affordability could be a reason for this difference [76], however many cardioprotective medications have a low cost (like aspirin), and in Argentina, Chile and Brazil, four of the medication classes (antiplatelets, beta-blockers ACEI, and statins) studied in this review are available free of charge [12,73,79]. Some studies remark that although medications may be affordable, not all of them are always available in the public sector [48,76,77,81]. The higher medication use in the higher SES patients may be explained by affordability and access to private healthcare [8,48,77,81].

#### Health care setting

Our meta-regression showed that health care setting was independently associated with the use of beta-blockers, antiplatelet drugs, aspirin and statin. In particular, in comparison to tertiary centers, the use of these medications was lower in primary care and community settings; use of antiplatelet drugs was lower in public centres; and use of aspirin was lower in cardiac rehabilitation settings. These findings are in line with previous results showing medication prevalence to be lower in studies conducted in primary care settings compared to those conducted in tertiary hospitals [8,59,81].

This may in part be due to overestimation of medication utilization prevalence in tertiary level clinical settings. First, patients attending tertiary centres may be older or more severely ill. Academic and tertiary hospitals have more capacity to provide active follow-up to patients compared to settings where care may be more fragmented. Therefore, patients attending academic or tertiary level centres for follow-up after an event may receive more specialized advice and prescriptions than patients attending a primary care facility, resulting higher rates of medication use [59,81]. Furthermore, it has been suggested that studies conducted in tertiary care settings may not include individuals without access to care [4]. Therefore, it is important to consider the setting of the study when interpreting results from research on prevalence of medication use.

### Strengths

This review summarizes evidence of a large number of studies from South America published in English, Spanish and Portuguese between 2000 and 2021. The comprehensive search included any article that contained data on any of the cardioprotective medication assessed in secondary prevention of CHD: anti-platelet drugs, lipid-lowering drugs, antihypertensive agents (beta-blockers, ACE-inhibitors, ARBs, diuretics, and nitrates), oral hypoglycaemics and insulin. We included articles from regional and international databases, and from a variety of settings (public and private centres, academic/tertiary centres and community settings, and urban and rural areas). In light of the results of this review, which show that care setting may influence the estimation of medication use prevalence, it is especially valuable that our review includes studies conducted in various settings.

In total, this review pooled the prevalence of cardioprotective medication use of 23,938 participants. Most of the articles included in this review had a low or moderate risk of bias, indicating low risk of bias for the pooled estimate.

### Limitations

This study aimed to summarize evidence on the prevalence of cardioprotective medication use for secondary prevention of CHD in South America. However, most studies were conducted in a limited number of countries and predominantly in Brazil, the largest and most populous country in South America. Our sensitivity analysis showed no prevalence differences between Brazil and the rest of the included countries, though no information was available for Bolivia, Ecuador, Guyana, Paraguay, Peru or Venezuela. As a result, the prevalence estimates of our study may not be generalizable for these countries. Furthermore, we were not able to run meta-regression for several medication classes because there were few observations. We pooled prevalence estimates from the included articles, but the heterogeneity in the from the meta-analysis models was high. We thus presented the results by subgroups and undertook meta-regression models to identify factors associated with medication use, which slightly reduced the heterogeneity of estimates.

## Conclusion

The current systematic review shows large variation in the use of cardioprotective medication among CHD patients in South America, ranging from 55.8% for the use of ACEI/ARB drugs to 85.1% for the use of aspirin. Medication use was lower in community settings, and it was often considered suboptimal in relation to clinical guidelines. The use of most cardioprotective medication classes has increased in the last decades though efforts should be made to further increase the use of these medications among CHD patients, particularly in community settings.

## Additional Files

The additional files for this article can be found as follows:

10.5334/gh.1124.s1 Appendix A. Figures 1 to 6.

10.5334/gh.1124.s2 Supplementary Files. Supplementary Files 1 to 3.
