# Supplementary material for: Prevalence of Cardioprotective Medication Use in Coronary Heart Disease Patients in South America: Systematic review and Meta-Analysis
Source: Glob Heart. 2022 Jun 8;17(1):37. doi: 10.5334/gh.1124 (PMC9187244; doi:10.5334/gh.1124)

Click here to access/download;Supplementary Files;Supplementary Figure 1.tif

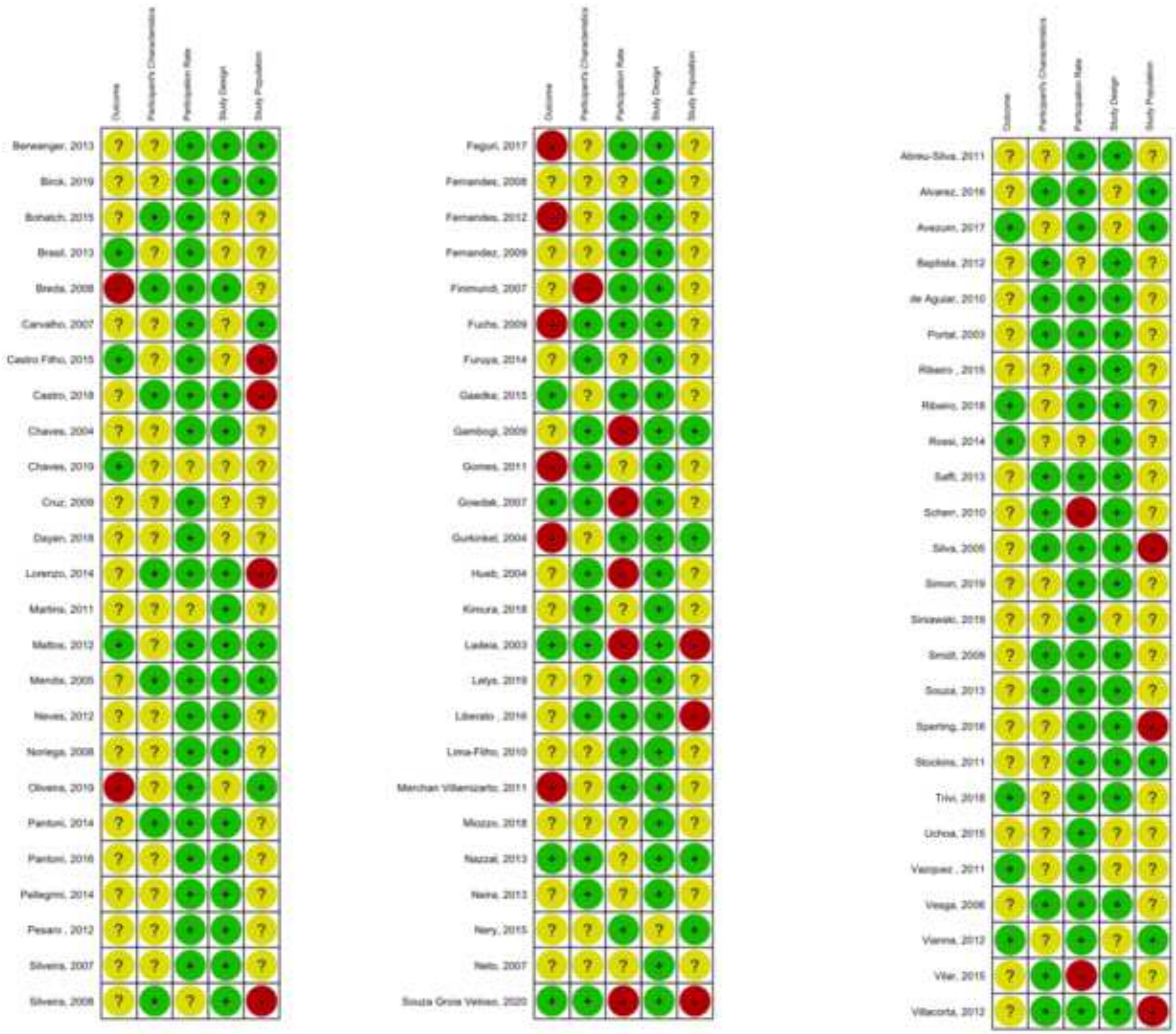

Click here to access/download;Supplementary Files;Supplementary Figure 2.png

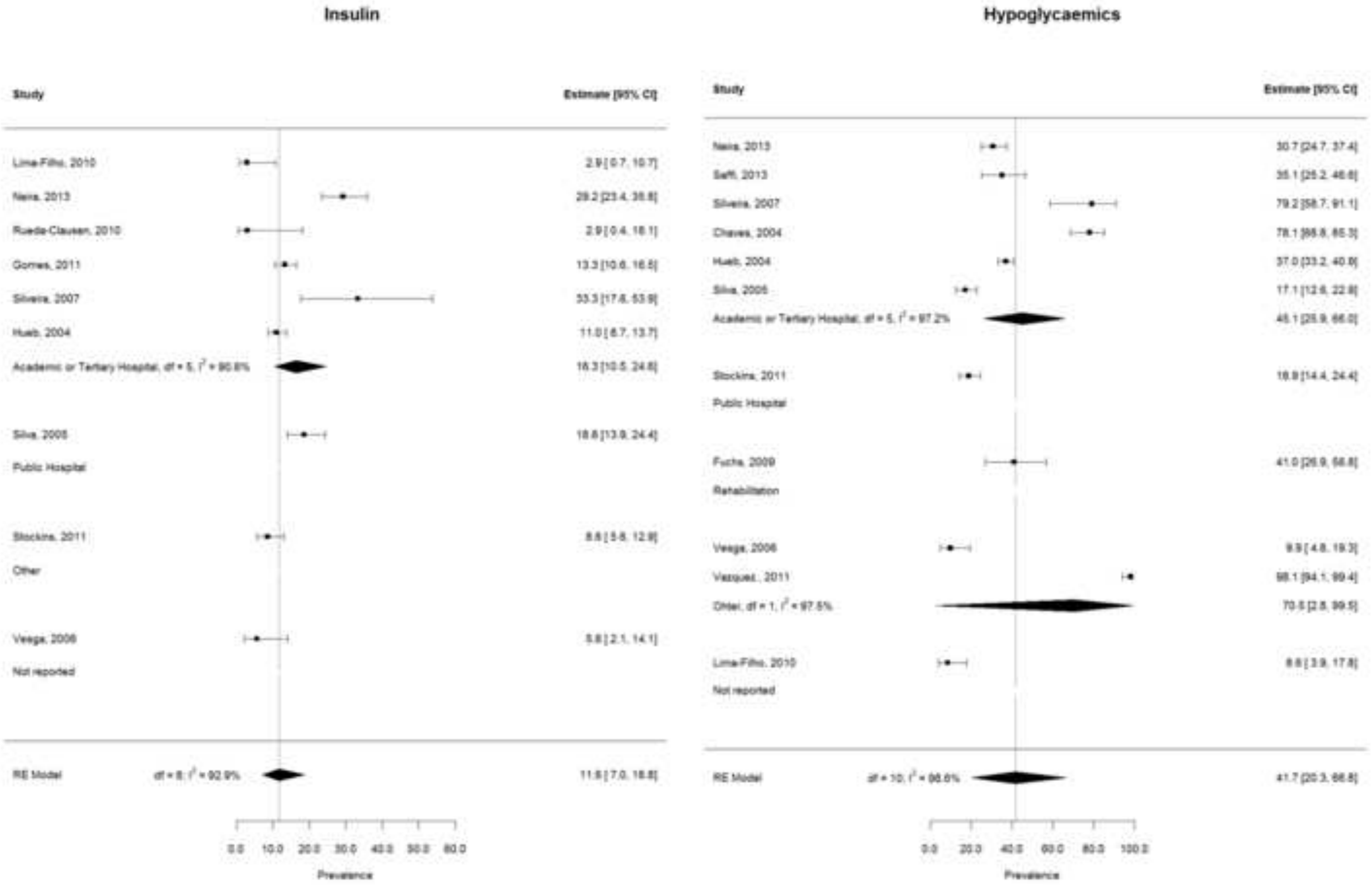

Click here to access/download;Supplementary Files;Supplementary Figure 3.tif

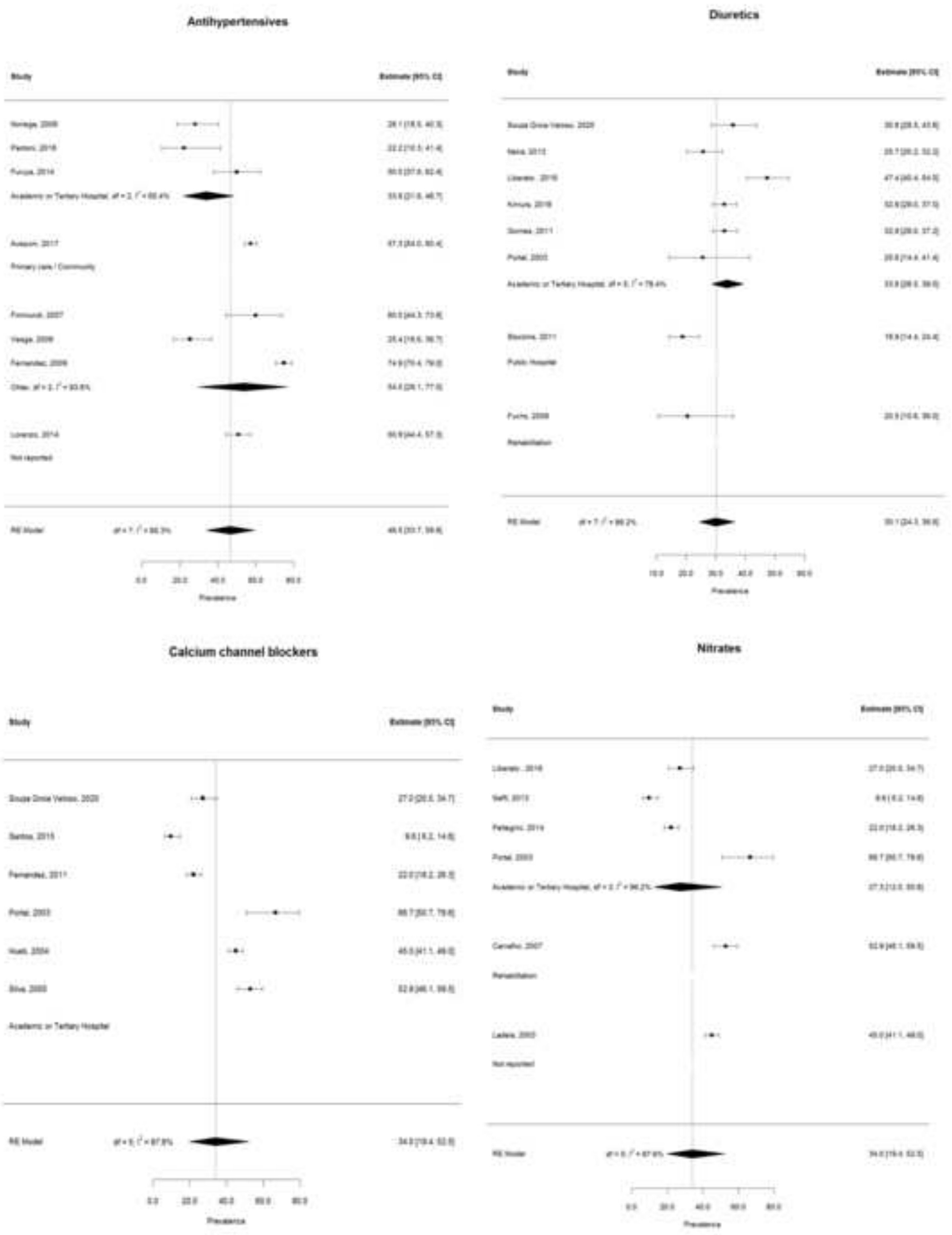

Supplementary Figure 4

Click here to access/download;Supplementary Files;Supplementary Figure 4.png

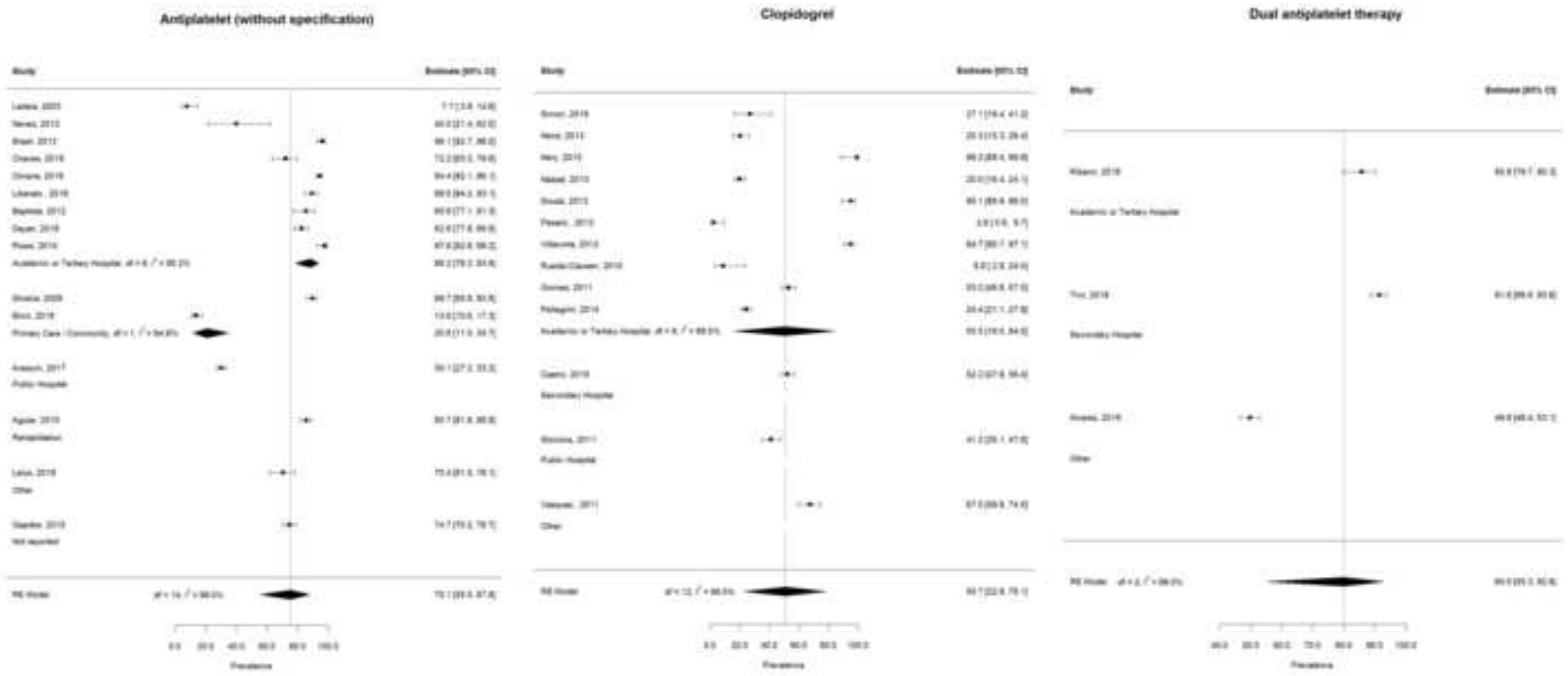

Click here to access/download;Supplementary Files;Supplementary Figure 5.png

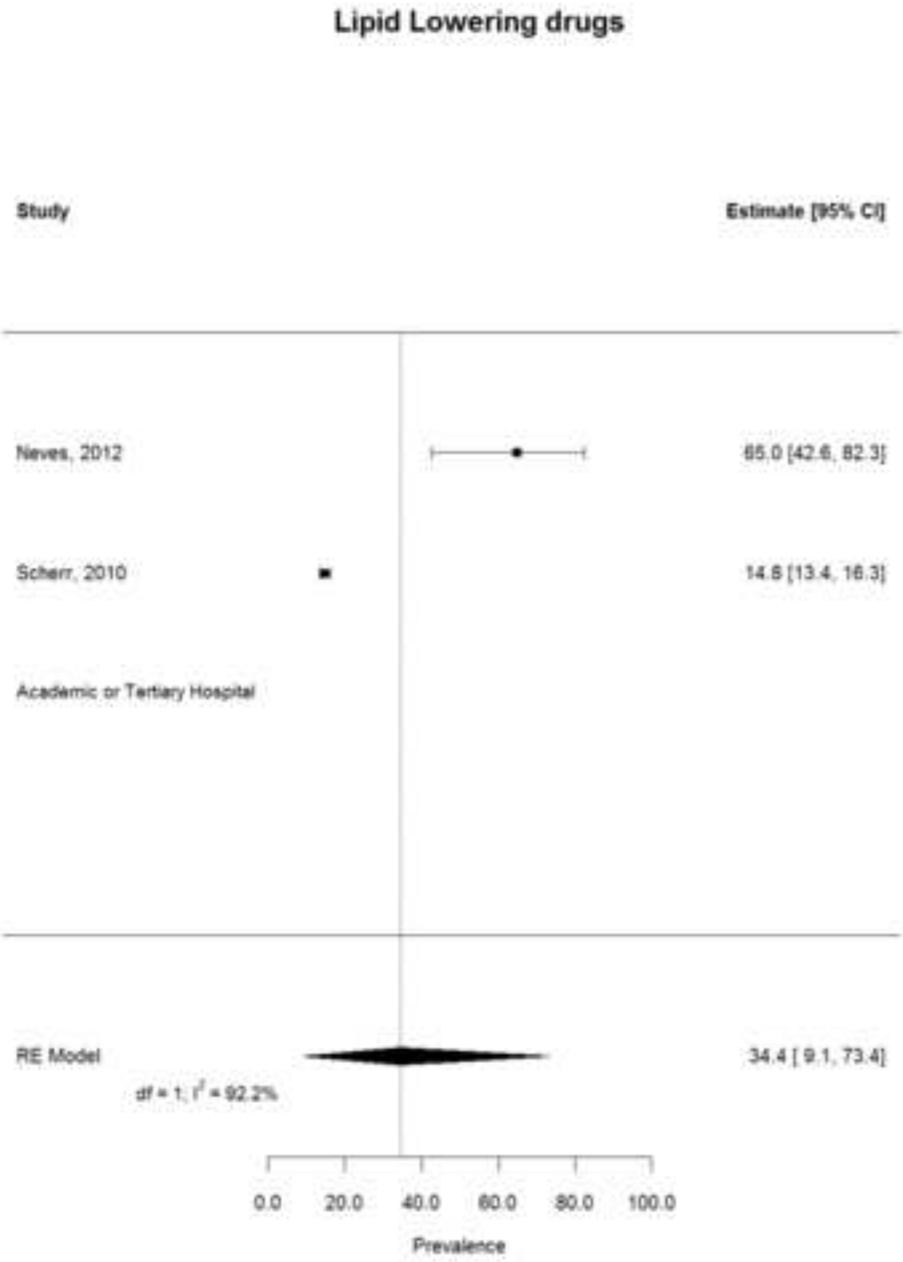

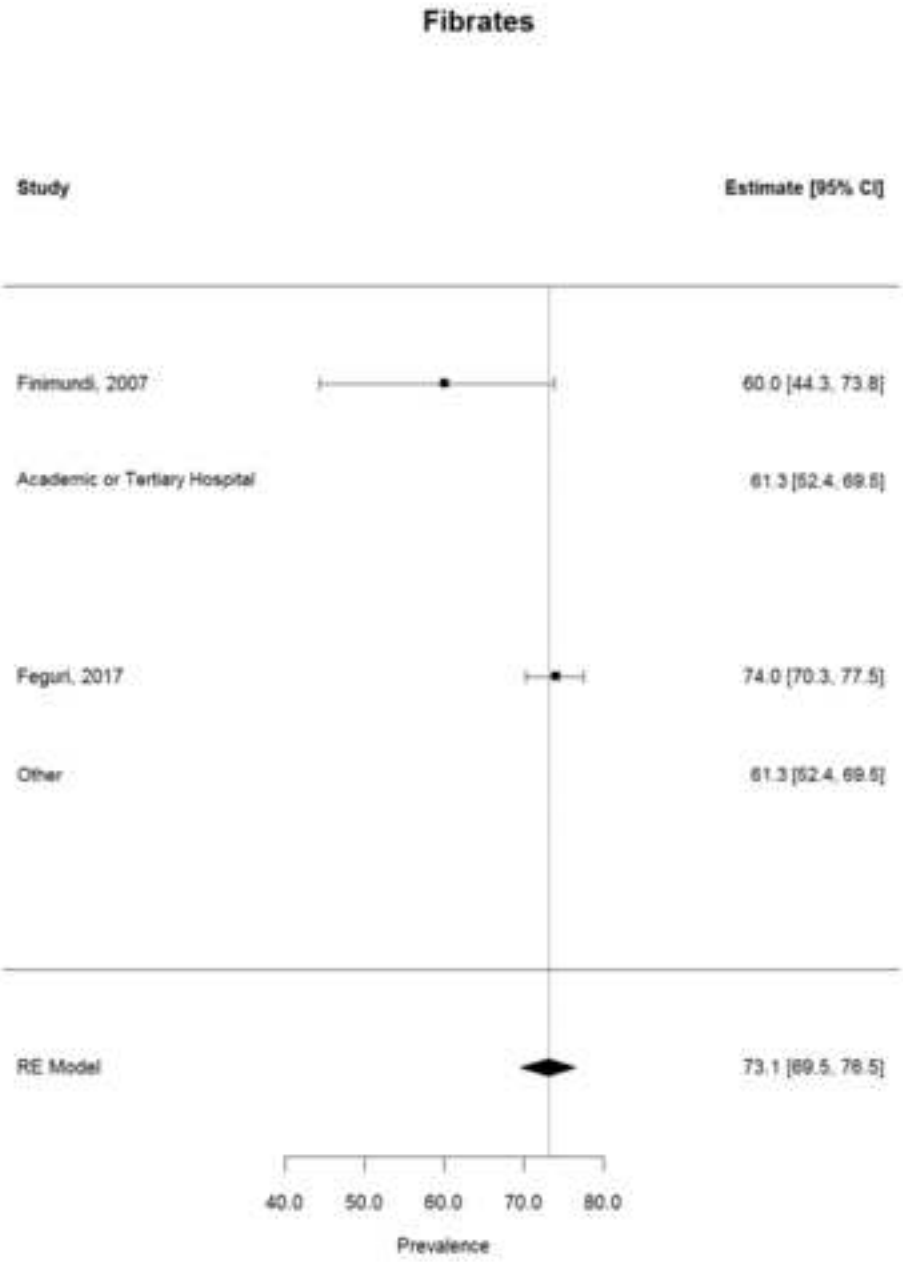

Click here to access/download;Supplementary Files;Supplementary Figure 6.tif

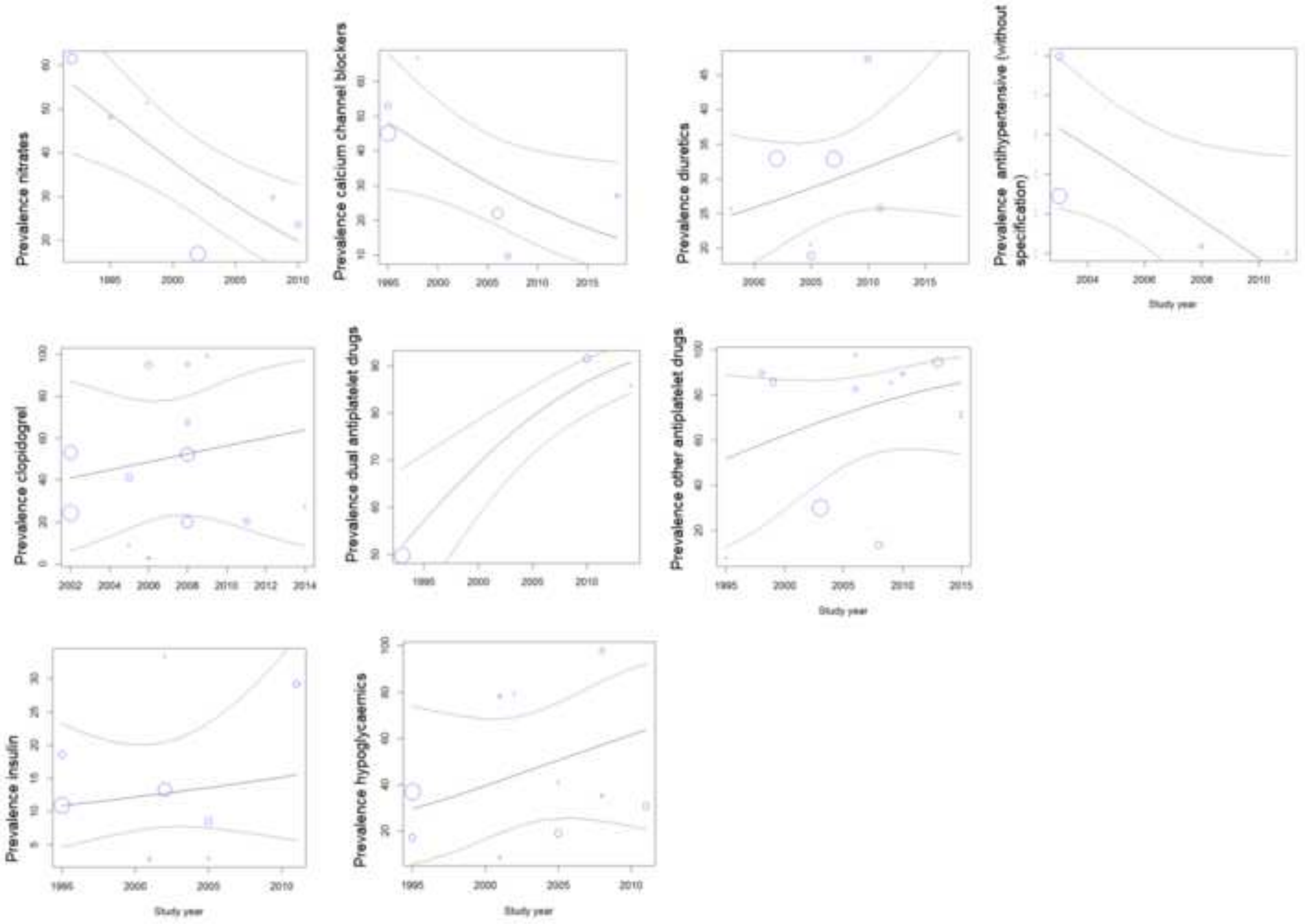

Supplement: Appendix A. — Figures 1 to 6. [file gh-17-1-1124-s1.pdf]
