# Supplementary material for: Prevalence of Cardioprotective Medication Use in Coronary Heart Disease Patients in South America: Systematic review and Meta-Analysis
Source: Glob Heart. 2022 Jun 8;17(1):37. doi: 10.5334/gh.1124 (PMC9187244; doi:10.5334/gh.1124)
Supplement: Supplementary Files. — Supplementary Files 1 to 3. [file gh-17-1-1124-s2.pdf]

Supplementary File 1: Prisma checklist for " Prevalence of cardioprotective medication use in patients with established coronary heart disease in South America: systematic review and meta-analysis".

PRISMA 2020 checklist

Section and Topic	Item	Checklist item	Location where item is reported
**TITLE**			
Title	1	Identify the report as a systematic review.	Page 1, line 2
**ABSTRACT**			
Abstract	2	See the PRISMA 2020 for Abstracts checklist.	Page 2 lines 2-17
**INTRODUCTION**			
Rationale	3	Describe the rationale for the review in the context of existing knowledge.	Page 3, lines 6-16
Objectives	4	Provide an explicit statement of the objective(s) or question(s) the review addresses.	Page 3, lines 17-21
**METHODS**			
Eligibility criteria	5	Specify the inclusion and exclusion criteria for the review and how studies were grouped for the syntheses.	Page 3 line 31- Page 4 line 6
Information sources	6	Specify all databases, registers, websites, organisations, reference lists and other sources searched or consulted to identify studies. Specify the date when each source was last searched or consulted.	Page 3 lines 28-29
Search strategy	7	Present the full search strategies for all databases, registers and websites, including any filters and limits used.	Supplementary File 2
Selection process	8	Specify the methods used to decide whether a study met the inclusion criteria of the review, including how many reviewers screened each record and each report retrieved, whether they worked independently, and if applicable, details of automation tools used in the process.	Page 4 lines 9-15
Data collection process	9	Specify the methods used to collect data from reports, including how many reviewers collected data from each report, whether they worked independently, any processes for obtaining or confirming data from study investigators, and if applicable, details of automation tools used in the process.	Page 4 lines 18-21.
Data items	10a	List and define all outcomes for which data were sought. Specify whether all results that were compatible with each outcome domain in each study were sought (e.g. for all measures, time points, analyses), and if not, the methods used to decide which results to collect.	Page 4 lines 30-39
	10b	List and define all other variables for which data were sought (e.g. participant and intervention characteristics, funding sources). Describe any assumptions made about any missing or unclear information.	Page 4 lines 21-29
Study risk of bias assessment	11	Specify the methods used to assess risk of bias in the included studies, including details of the tool(s) used, how many reviewers assessed each study and whether they worked independently, and if applicable, details of automation tools used in the process.	Page 5 lines 1-11.
Effect measures	12	Specify for each outcome the effect measure(s) (e.g. risk ratio, mean difference) used in the synthesis or presentation of results.	Page 5 lines 14-15 and 28-30
Synthesis	13a	Describe the processes used to decide which studies were eligible for each synthesis (e.g. tabulating the study intervention	Page 5 lines 15-20

Section and Topic	Item	Checklist item	Location where item is reported
methods		characteristics and comparing against the planned groups for each synthesis (item #5)).	
	13b	Describe any methods required to prepare the data for presentation or synthesis, such as handling of missing summary statistics, or data conversions.	Page 4 lines 34-35
	13c	Describe any methods used to tabulate or visually display results of individual studies and syntheses.	Page 5 lines 14-15, 21-23, 28-30.
	13d	Describe any methods used to synthesize results and provide a rationale for the choice(s). If meta-analysis was performed, describe the model(s), method(s) to identify the presence and extent of statistical heterogeneity, and software package(s) used.	Page 5 lines 15-20, 21-24, 27-28.
	13e	Describe any methods used to explore possible causes of heterogeneity among study results (e.g. subgroup analysis, meta-regression).	Page 5 lines 31-37
	13f	Describe any sensitivity analyses conducted to assess robustness of the synthesized results.	Page 5 24-26.
Reporting bias assessment	14	Describe any methods used to assess risk of bias due to missing results in a synthesis (arising from reporting biases).	NA
Certainty assessment	15	Describe any methods used to assess certainty (or confidence) in the body of evidence for an outcome.	Page 5, lines 22-24
**RESULTS**			
Study selection	16a	Describe the results of the search and selection process, from the number of records identified in the search to the number of studies included in the review, ideally using a flow diagram.	Page 6 lines 3-9, Figure 1.
	16b	Cite studies that might appear to meet the inclusion criteria, but which were excluded, and explain why they were excluded.	NA
Study characteristics	17	Cite each included study and present its characteristics.	Table 1, Citations in pages 12-18.
Risk of bias in studies	18	Present assessments of risk of bias for each included study.	Supplementary Figure 1
Results of individual studies	19	For all outcomes, present, for each study: (a) summary statistics for each group (where appropriate) and (b) an effect estimate and its precision (e.g. confidence/credible interval), ideally using structured tables or plots.	Figures 3-6
Results of syntheses	20a	For each synthesis, briefly summarise the characteristics and risk of bias among contributing studies.	Figures 3-6
	20b	Present results of all statistical syntheses conducted. If meta-analysis was done, present for each the summary estimate and its precision (e.g. confidence/credible interval) and measures of statistical heterogeneity. If comparing groups, describe the direction of the effect.	Page 7 lines 9-26, 28-33, 36-40, Page 8 lines 30-40, Figures 3-6, Table 2
	20c	Present results of all investigations of possible causes of heterogeneity among study results.	Page 8 lines 30-40, Table 2
	20d	Present results of all sensitivity analyses conducted to assess the robustness of the synthesized results.	Page 7 line s20-21
Reporting biases	21	Present assessments of risk of bias due to missing results (arising from reporting biases) for each synthesis assessed.	NA
Certainty of	22	Present assessments of certainty (or confidence) in the body of evidence for each outcome assessed.	Page 6 lines 18-

Section and Topic	Item	Checklist item	Location where item is reported
evidence			21, Figures 3-6, Table 2
**DISCUSSION**			
Discussion	23a	Provide a general interpretation of the results in the context of other evidence.	Page 9 line 13 – age 11 line 23
	23b	Discuss any limitations of the evidence included in the review.	Page 12 lines 1-9
	23c	Discuss any limitations of the review processes used.	Page 12 lines 10-13.
	23d	Discuss implications of the results for practice, policy, and future research.	Page 12 lines 19-22.
**OTHER INFORMATION**			
Registration and protocol	24a	Provide registration information for the review, including register name and registration number, or state that the review was not registered.	Page 3 line 26
	24b	Indicate where the review protocol can be accessed, or state that a protocol was not prepared.	Supplementary File 2
	24c	Describe and explain any amendments to information provided at registration or in the protocol.	NA
Support	25	Describe sources of financial or non-financial support for the review, and the role of the funders or sponsors in the review.	NA
Competing interests	26	Declare any competing interests of review authors.	NA
Availability of data, code and other materials	27	Report which of the following are publicly available and where they can be found: template data collection forms; data extracted from included studies; data used for all analyses; analytic code; any other materials used in the review.	Supplementary File 2 (search strategy)

Supplementary File 2: Prospero registration and search strategy used in Pubmed, Embase, Cochrane, LILACs and SciELO

This review was registered with PROSPERO (registration number CRD42020206657), accessible at https://www.crd.york.ac.uk/prospero/display_record.php?ID=CRD42020206657.

Pubmed

("CHD"[Title/Abstract] OR "coronary heart disease*"[Title/Abstract] OR "CAD"[Title/Abstract] OR "coronary artery disease*"[Title/Abstract] OR "coronary disease*"[Title/Abstract] OR "myocardial infarction*"[Title/Abstract] OR "MI"[Title/Abstract] OR "angina pectoris"[Title/Abstract] OR "percutaneous intervention*"[Title/Abstract] OR "STEMI"[Title/Abstract] OR "NSTEMI"[Title/Abstract] OR "acute coronary syndrom*"[Title/Abstract] OR "ACS"[Title/Abstract] OR "CABG"[Title/Abstract] OR "coronary artery bypass graft*"[Title/Abstract] OR "Coronary Arterioscleros*"[Title/Abstract] OR "Coronary Atheroscleroses"[Title/Abstract] OR "Coronary Artery Disease"[Mesh])

AND

("medication"[Title/Abstract] OR "secondary prevention"[Title/Abstract] OR "treatment"[Title/Abstract] OR "drug*"[Title/Abstract] OR "medicines"[Title/Abstract] OR "cardiac rehab*"[Title/Abstract]
OR "anti-platelet"[Title/Abstract] OR "platelet aggregation inhibitor*"[Title/Abstract] OR "platelet antiaggregant*"[Title/Abstract] OR "platelet inhibitor*"[Title/Abstract] OR "Platelet Aggregation Inhibitors"[Mesh]
OR "statin*"[Title/Abstract] OR "anticholesterolemic*"[Title/Abstract] OR "hypocholesterolemic*"[Title/Abstract] OR "cholesterol inhibitors*"[Title/Abstract] OR "Hydroxymethylglutaryl CoA Reductase Inhibitor*"[Title/Abstract] OR "HMG CoA reductase inhibitors*"[Title/Abstract] OR "lipid-lowering"[Title/Abstract] OR "hypolipidemic*"[Title/Abstract] OR "Antihyperlipidemics*"[Title/Abstract] OR "antilipemic*"[Title/Abstract] OR "Hypolipidemic Agents"[Mesh]
OR "beta-blocker*"[Title/Abstract] OR "beta Antagonist*"[Title/Abstract] OR "Adrenergic beta-Antagonists"[MeSH] OR "Ca antagonist"[Title/Abstract] OR "Calcium antagonist"[Title/Abstract] OR "calcium channel block*"[Title/Abstract] OR "calcium channel antagonist*"[Title/Abstract] OR "calcium inhibitor*"[Title/Abstract] OR "calcium block*"[Title/Abstract] OR "Calcium Channel Blockers"[Mesh] OR "ACE-I"[Title/Abstract] OR "ACE-inhibitor"[Title/Abstract] OR "angiotensin converting enzyme inhibitor*"[Title/Abstract] OR "kininase II inhibitor*"[Title/Abstract] OR "kininase II antagonist*"[Title/Abstract] OR "Angiotensin I Converting Enzyme Inhibitor*"[Title/Abstract] OR "Angiotensin I Converting Enzyme antagonist*"[Title/Abstract] OR "Angiotensin Converting Enzyme antagonist*"[Title/Abstract] OR "Angiotensin-Converting Enzyme Inhibitors"[Mesh] OR "anti-hypertensive*"[Title/Abstract] OR "Antihypertensive Agents"[MeSH] OR "diuretic*"[Title/Abstract] OR "Diuretics"[MeSH]
OR "hypoglycaemic*"[Title/Abstract] OR "hypoglycemic*"[Title/Abstract] OR "antihyperglycemic*"[Title/Abstract]
OR "nitrate*"[Title/Abstract] OR "dinitrate*"[Title/Abstract] OR "Isosorbide Dinitrate"[Mesh] OR "isosorbide mononitrate"[Title/Abstract] OR "isosorbide-5-mononitrate"[Supplementary Concept] OR "nitroglycerin"[Title/Abstract] OR "glyceryl trinitrate"[Title/Abstract] OR "nitroglycerin"[Mesh]
OR "insulin"[Title/Abstract] OR "insulin"[Mesh])

AND

("South America"[Mesh] OR "South America"[TW] OR "Latin America"[TW] OR "Caribbean"[TW] OR "Paraguay"[TW] OR "Argentina"[TW] OR "Peru"[TW] OR "Ecuador"[TW] OR "Bolivia"[TW] OR "Brazil"[TW] OR "Brasil"[TW] OR "Suriname"[TW] OR "Chile"[TW] OR "Colombia" OR "Uruguay"[TW] OR "Venezuela"[TW] OR "French Guiana"[tw] OR "Guyana"[tw]) AND 2000:2020 [dp] AND ((humans[Filter]) AND (dutch[Filter] OR english[Filter] OR french[Filter] OR portuguese[Filter] OR spanish[Filter]))

EMBASE

('chd':ti,ab,kw OR 'coronary heart disease*':ti,ab,kw OR 'cad':ti,ab,kw OR 'coronary artery disease*':ti,ab,kw OR 'coronary disease*':ti,ab,kw OR 'myocardial infarction*':ti,ab,kw OR 'mi':ti,ab,kw OR 'angina pectoris':ti,ab,kw OR 'percutaneous intervention*':ti,ab,kw OR 'stemi':ti,ab,kw OR 'nstemi':ti,ab,kw OR 'acute coronary syndrom*':ti,ab,kw OR 'acs':ti,ab,kw OR 'cabg':ti,ab,kw OR 'coronary artery bypass graft*':ti,ab,kw OR 'coronary arterioscleros*':ti,ab,kw OR 'coronary atheroscleroses':ti,ab,kw OR 'coronary artery disease'/exp)

AND

('medication':ti,ab,kw OR 'secondary prevention':ti,ab,kw OR 'treatment':ti,ab,kw OR 'drug*':ti,ab,kw OR 'medicines':ti,ab,kw OR 'cardiac rehab*':ti,ab,kw OR 'anti-platelet':ti,ab,kw OR 'platelet aggregation inhibitor*':ti,ab,kw OR 'platelet antiaggregant*':ti,ab,kw OR 'platelet inhibitor*':ti,ab,kw OR 'anticoagulant agent'/exp OR 'statin*':ti,ab,kw OR 'anticholesterolemic*':ti,ab,kw OR 'hypocholesterolemic*':ti,ab,kw OR 'cholesterol inhibitors*':ti,ab,kw OR 'hydroxymethylglutaryl coa reductase inhibitor*':ti,ab,kw OR 'hmg coa reductase inhibitors*':ti,ab,kw OR 'lipid-lowering':ti,ab,kw OR 'hypolipidemic*':ti,ab,kw OR 'antihyperlipidemics*':ti,ab,kw OR 'antilipemic*':ti,ab,kw OR 'antilipemic agent'/exp OR 'beta-blocker*':ti,ab,kw OR 'beta antagonist*':ti,ab,kw OR 'ca antagonist':ti,ab,kw OR 'calcium antagonist':ti,ab,kw OR 'calcium channel block*':ti,ab,kw OR 'calcium channel antagonist*':ti,ab,kw OR 'calcium inhibitor*':ti,ab,kw OR 'calcium block*':ti,ab,kw OR 'ace-i':ti,ab,kw OR 'ace-inhibitor':ti,ab,kw OR 'angiotensin converting enzyme inhibitor*':ti,ab,kw OR 'kininase ii inhibitor*':ti,ab,kw OR 'kininase ii antagonist*':ti,ab,kw OR 'angiotensin i converting enzyme inhibitor*':ti,ab,kw OR 'angiotensin i converting enzyme antagonist*':ti,ab,kw OR 'angiotensin converting enzyme antagonist*':ti,ab,kw OR 'anti-hypertensive*':ti,ab,kw OR 'antihypertensive agent'/exp OR 'diuretic*':ti,ab,kw OR 'diuretic agent'/exp OR 'hypoglycaemic*':ti,ab,kw OR 'hypoglycemic*':ti,ab,kw OR 'antihyperglycemic*':ti,ab,kw OR 'nitrate*':ti,ab,kw OR 'dinitrate*':ti,ab,kw OR 'isosorbide mononitrate':ti,ab,kw OR 'nitroglycerin':ti,ab,kw OR 'glyceryl trinitrate':ti,ab,kw OR 'insulin':ti,ab,kw OR 'insulin'/exp)

AND

('south america'/exp OR 'south america':ti,ab,ca,kw OR 'latin america':ti,ab,ca,kw OR 'caribbean':ti,ab,ca,kw OR 'paraguay':ti,ab,ca,kw OR 'argentina':ti,ab,ca,kw OR 'peru':ti,ab,ca,kw OR 'ecuador':ti,ab,ca,kw OR 'bolivia':ti,ab,ca,kw OR 'brazil':ti,ab,ca,kw OR 'brasil':ti,ab,ca,kw OR 'suriname':ti,ab,ca,kw OR 'chile':ti,ab,ca,kw OR 'colombia' OR 'uruguay':ti,ab,ca,kw OR 'venezuela':ti,ab,ca,kw OR 'french guiana':ti,ab,ca,kw OR 'guyana':ti,ab,ca,kw) AND (english:la OR dutch:la OR spanish:la OR french:la OR portuguese:la)

AND [2000-2020]/py AND [embase]/lim

NOT ('article in press'/it OR 'chapter'/it OR 'conference abstract'/it OR 'conference paper'/it OR 'conference review'/it OR 'editorial'/it OR 'letter'/it OR 'note'/it OR 'review'/it OR 'short survey'/it)

NOT ('animal experiment'/de OR 'animal model'/de OR 'animal tissue'/de OR 'case report'/de OR 'clinical protocol'/de OR 'human cell'/de OR 'human tissue'/de OR 'in vitro study'/de OR 'interview'/de OR 'meta analysis'/de OR 'meta analysis topic'/de OR 'nonhuman'/de)

NOT ([adolescent]/lim OR [child]/lim OR [embryo]/lim OR [fetus]/lim OR [infant]/lim OR [newborn]/lim OR [preschool]/lim OR [school]/lim)

Cochrane
Link to search: https://www.cochranelibrary.com/advanced-search/search-manager?search=3768067

#1 ("CHD" OR "coronary heart disease*" OR "CAD" OR "coronary artery disease*" OR "coronary disease*" OR "myocardial infarction*" OR "MI" OR "angina pectoris" OR "percutaneous intervention*" OR "STEMI" OR "NSTEMI" OR "acute coronary syndrom*" OR "ACS" OR "CABG" OR "coronary artery bypass graft*" OR "Coronary Arterioscleros*" OR "Coronary Atheroscleroses"):ti,ab,kw (Word variations have been searched)

#2 ("medication" OR "secondary prevention" OR "treatment" OR "drug*" OR "medicin*" OR "cardiac rehab*" OR "anti platelet" OR "platelet aggregation inhibitor*" OR "platelet antiaggregant*" OR "platelet inhibitor*" OR "statin*" OR "anticholesterolemic*" OR "hypocholesterolemic*" OR "cholesterol inhibitors*" OR "Hydroxymethylglutaryl CoA Reductase Inhibitor*" OR "HMG CoA reductase inhibitors*" OR "lipid lowering" OR "hypolipidemic*" OR "Antihyperlipidemics*" OR "antilipemic*" OR "beta blocker*" OR "beta Antagonist*" OR "Ca antagonist" OR "Calcium antagonist" OR "calcium channel block*" OR "calcium channel antagonist*" OR "calcium inhibitor*" OR "calcium block*" OR "ACE I" OR "ACE-inhibitor" OR "angiotensin converting enzyme inhibitor*" OR "kininase II inhibitor*" OR "kininase II antagonist*" OR "Angiotensin I Converting Enzyme Inhibitor*" OR "Angiotensin I Converting Enzyme antagonist*" OR "Angiotensin Converting Enzyme antagonist*" OR "anti-hypertensive*" OR "diuretic*" OR "hypoglycaemic*" OR "hypoglycemic*" OR "antihyperglycemic*" OR "nitrate*" OR "dinitrate*" OR "isosorbide mononitrate" OR "isosorbide-5-mononitrate" OR "nitroglycerin" OR "glyceryl trinitrate" OR "insulin"):ti,ab,kw (Word variations have been searched)

#3 "South America" OR "Latin America" OR "Caribbean" OR "Paraguay" OR "Argentina" OR "Peru" OR "Ecuador" OR "Bolivia" OR "Brazil" OR "Brasil" OR "Suriname" OR "Chile" OR "Colombia" OR "Uruguay" OR "Venezuela" OR "French Guiana" OR "Guyana"

#4 MeSH descriptor: [Coronary Artery Disease] explode all trees

#5 MeSH descriptor: [Platelet Aggregation Inhibitors] explode all trees
#6 MeSH descriptor: [Hypolipidemic Agents] explode all trees
#7 MeSH descriptor: [Antihypertensive Agents] explode all trees
#8 MeSH descriptor: [Diuretics] explode all trees
#9 MeSH descriptor: [Isosorbide Dinitrate] explode all trees
#10 MeSH descriptor: [Insulins] explode all trees

#11 MeSH descriptor: [South America] explode all trees

#12 (#1 OR #4) AND (#2 OR #5 OR #6 OR #7 OR #8 OR #9 OR #10) AND (#3 OR #11)
Searched with Cochrane Library publication date Between Jan 2000 and Jul 2020, in Trials (Word variations have been searched)

LILACS:

(MH:C14.280.647.250.260$ OR MH:C14.907.137.126.339$ OR MH:C14.907.585.250.260$ OR chd OR "coronary heart disease" OR CAD OR "coronary artery disease" OR "coronary disease" OR "myocardial infarction" OR mi OR "angina pectoris" OR "percutaneous intervention" OR stemi OR nstemi OR acs OR cabg OR "arteriosclerosis" OR "atherosclerosis" OR "coronary syndrome")

AND

(Medication$ OR "secondary prevention" OR treatment OR drug OR medicines OR "cardiac rehabilitation" OR "anti platelet" OR statin$ OR hypolipidemic OR "beta blocker" OR "calcium inhibitor" OR "ACE I" OR "ACE inhibitor" OR antihypertensive$ OR diuretic OR hypoglycaemic OR hypoglycemic OR nitrate OR insulin OR "ant hypertensive" OR antihyperglycemic OR dinitrate OR nitroglycerin OR MH:D27.505.519.186.071$ OR MH:D27.505.954.557.500$ OR MH: D27.505.954.411.162$ OR MH: D27.505.696.422$)

AND (fulltext:("1") AND db:("LILACS"))

AND (year_cluster:[2000 TO 2020])

AND (type:(article))
AND NOT type_of_study:(health_economic_evaluation OR case_reports OR guideline OR systematic_reviews)

AND limit:("humans")
AND NOT limit:(animals OR adolescent OR child OR "child, preschool" OR infant OR newborn)

DECS Used in search:

- Myocardial Ischemia: C14.280.647, C14.907.585
- Coronary artery disease: C14.280.647.250.260, C14.907.137.126.339, C14.907.585.250.260
- Hypolipidemic Agents: D27.505.519.186.071, D27.505.954.557.500
- “Antihypertensive Agents”: D27.505.954.411.162
- Hypoglycemic agents: D27.505.696.422

Terms left out because they count as ‘AND’:

- OR “coronary bypass graft” OR “platelet aggregation inhibitor" OR “platelet antiaggregant" OR “platelet inhibitor" OR “cholesterol inhibitors" antilipemic OR OR “beta Antagonist" OR “Ca antagonist”OR “calcium channel block" OR “calcium channel antagonist”

Also searhable by pasting this in the bar (IAHx):

((mh:c14.280.647.250.260* OR mh:c14.907.137.126.339* OR mh:c14.907.585.250.260* OR chd OR "coronary heart disease" OR cad OR "coronary artery disease" OR "coronary disease" OR "myocardial infarction" OR mi OR "angina pectoris" OR "percutaneous intervention" OR stemi OR nstemi OR acs OR cabg OR "arteriosclerosis" OR "atherosclerosis" OR "coronary syndrome") AND (medication* OR "secondary prevention" OR treatment OR drug OR medicines OR "cardiac rehabilitation" OR "anti platelet" OR statin* OR hypolipidemic OR "beta blocker" OR "calcium inhibitor" OR "ACE I" OR "ACE inhibitor" OR antihypertensive* OR diuretic OR hypoglycaemic OR hypoglycemic OR nitrate OR insulin OR "ant hypertensive" OR antihyperglycemic OR dinitrate OR nitroglycerin OR mh:d27.505.519.186.071* OR mh:d27.505.954.557.500* OR mh: d27.505.954.411.162* OR mh: d27.505.696.422*) AND (fulltext:("1") AND db:("LILACS")) AND (year_cluster:[2000 TO 2020]) AND (type:(article)) NOT type_of_study:(health_economic_evaluation OR case_reports OR guideline OR systematic_reviews) AND limit:("humans") NOT limit:(animals OR adolescent OR child OR "child, preschool" OR infant OR newborn)) AND (db:("LILACS"))

SCIELO:
subject:(chd OR "coronary heart disease" OR cad OR "coronary artery disease" OR "coronary disease" OR "myocardial infarction" OR mi OR "angina pectoris" OR "percutaneous intervention" OR stemi OR nstemi OR acs OR cabg OR "arteriosclerosis" OR "atherosclerosis" OR "coronary syndrome")

AND

subject:(medication* OR "secondary prevention" OR treatment OR drug OR medicines OR "cardiac rehabilitation" OR "anti platelet" OR statin* OR hypolipidemic OR "beta blocker" OR "calcium inhibitor" OR "ACE I" OR "ACE inhibitor" OR antihypertensive* OR diuretic OR hypoglycaemic OR hypoglycemic OR nitrate OR insulin OR "ant hypertensive" OR antihyperglycemic OR dinitrate OR nitroglycerin)

AND

year_cluster:("2000" OR "2001" OR "2002" OR "2003" OR "2004" OR "2005" OR "2006" OR "2007" OR "2008" OR "2009" OR "2010" OR "2011" OR "2012" OR "2013" OR "2014" OR "2015" OR "2016" OR "2017" OR "2018" OR "2019" OR "2020")

AND

type:("research-article")

AND NOT

in:("esp" OR "mex" OR "cub" OR "cri" OR "sza" OR "prt")

Supplementary File 3

Supplementary File 3 A: Tool for risk of bias assessment (Adapted from Zhao et al, 2016).

Bias type	Low risk	Moderate risk	High risk
**Study design**	• Prospective data collection (clinical assessment)	• Retrospective data collection (medical records or self-reported questionnaire/survey)	• Unclear data collection and statistical analysis
**Study population**	• Specific and detailed sample selection criteria<br>• Sample from general population but not selected group with multiple centres<br>• Specific and detailed sample selection criteria	• Sample selected from large population but selection criteria not defined<br>• Sample selection ambiguous but may be representative;<br>• Analysis to adjust for sampling strategy bias	• Unclear sample selection criteria<br>• Sample is selected from single centre and not representative
**Participant rate**	• High participant rate (>85%)	• Moderate participant rate (70-85%)	• Low participant rate (<70%)
**Participants' characteristics**	• Diagnosis using consistent criteria and direct examination<br>• Specific and detailed recruitment time period<br>• Consecutive participants >18 years old<br>• Specific and detailed determinants (e.g. age)	• Diagnosis assessment from medical records, questionnaire, survey, administrative database or register<br>• Wide and undetailed recruitment time period<br>• Specific and detailed determinants	• Diagnosis assessment from non-validated data or generic estimate from overall population<br>• Unknown performed time or location<br>• No determinants information available
**Outcomes**	• Detailed information on prevalence of cardiovascular medications usage<br>• Detailed information on absolute level of blood pressure, lipids, and glucose	• Detailed information on prevalence of cardiovascular medications usage	• Unclear outcome data or data collection methodology

Supplementary File 3 B: Results of the risk of bias assessment of all publications that resulted from the screening phase of the review.

Publication	Study Design	Study population	Participation rate	Participants characteristics	Outcome	Risk of bias
Mattos, 2012	2	2	2	1	2	9
Mendis, 2005	2	2	2	2	1	9
Nazzal, 2013	2	2	1	2	2	9
Abreu-Silva, 2011	2	1	2	1	2	8
Aguiar, 2010	2	1	2	2	1	8
Alvarez, 2016	1	2	2	2	1	8
Avezum, 2017	1	2	2	1	2	8
Berwanger, 2013	2	2	2	1	1	8
Birck, 2019	2	2	2	1	1	8
Gaedke, 2015	2	1	2	1	2	8
Pantoni, 2014	2	1	2	2	1	8
Portal, 2003	2	1	2	2	1	8
Ribeiro, 2018	2	1	2	1	2	8
Saffi, 2013	2	1	2	2	1	8
Smidt, 2009	2	1	2	2	1	8
Souza, 2013	2	1	2	2	1	8
Stockins, 2011	2	2	2	1	1	8

Trivi, 2018	2	1	2	1	2	8
Vesga, 2006	2	1	2	2	1	8
Vianna, 2012	1	2	2	1	2	8
Baptista, 2012	2	1	1	2	1	7
Bohatch, 2015	1	1	2	2	1	7
Brasil, 2013	1	1	2	1	2	7
Breda, 2008	2	1	2	2	0	7
Carvalho, 2007	1	2	2	1	1	7
Castro, 2018	2	0	2	2	1	7
Chaves, 2004	2	1	2	1	1	7
Dayan, 2018	1	1	2	1	2	7
Fernandez, 2009	2	1	2	1	1	7
Finimundi, 2007	2	1	2	0	2	7
Fuchs, 2009	2	1	2	2	0	7
Furuya, 2014	2	1	1	2	1	7
Gambogi, 2009	2	2	0	2	1	7
Gowdak, 2007	2	1	0	2	2	7
Gurfinkel, 2004	2	2	2	1	0	7
Hueb, 2004	2	1	0	2	2	7
Kimura, 2018	2	1	1	2	1	7
Lelys, 2019	2	1	2	1	1	7
Liberato , 2016	2	0	2	2	1	7
Lima-Filho, 2010	2	1	2	1	1	7
Lorenzo, 2014	2	0	2	2	1	7
Neira, 2013	2	1	1	2	1	7
Nery, 2015	1	2	2	1	1	7
Neves, 2012	2	1	2	1	1	7
Noriega, 2008	2	1	2	1	1	7
Pantoni, 2016	2	1	2	1	1	7
Pellegrini, 2014	2	1	2	1	1	7
Pesaro , 2012	2	1	2	1	1	7
Ribeiro , 2015	2	1	2	1	1	7
Rossi, 2014	2	1	1	1	2	7
Silva, 2005	2	0	2	2	1	7
Silveira, 2007	2	1	2	1	1	7
Silveira, 2008	2	0	1	2	2	7
Simon, 2019	2	1	2	1	1	7
Siniawski, 2019	1	1	2	1	2	7
Uchoa, 2015	1	1	2	1	2	7
Vazquez , 2011	1	1	2	1	2	7
Vilar, 2015	2	1	0	2	2	7
Villacorta, 2012	2	0	2	2	1	7
Castillo y Costa, 2018	2	0	0	2	2	6
Chaves, 2019	1	1	1	1	2	6
Cruz, 2009	1	1	2	1	1	6
Feguri, 2017	2	1	2	1	0	6
Fernandes, 2012	2	1	2	1	0	6
Garlet, 2017	1	1	1	1	2	6
Gomes, 2011	2	1	1	2	0	6

Ladeia, 2003	2	0	0	2	2	6
Merchan Villamizarto, 2011	2	1	2	1	0	6
Oliveira, 2019	1	2	2	1	0	6
Rueda-Clausen, 2010	2	0	1	1	2	6
Santos, 2015	2	1	0	1	2	6
Scherr, 2010	2	1	0	2	1	6
Souza Groia Veloso, 2020	2	0	0	2	2	6
Barbosa, 2014	2	1	1	1	0	5
Blanco MI, 2016	1	0	2	2	0	5
Burdiat, 2016	2	0	2	1	0	5
Cardozo, 2015	2	1	0	0	2	5
Castello Simoes, 2015	1	1	1	2	0	5
Cesena, 2004	0	0	2	1	2	5
Duque, 2018	2	0	1	2	0	5
Fantin, 2011	2	1	1	1	0	5
Feguri, 2012	2	1	0	2	0	5
Fernandes, 2006	2	1	1	1	0	5
Fernandes, 2008	2	1	1	1	0	5
Ferreira, 2017	1	1	2	1	0	5
Lombo, 2010	2	0	1	1	1	5
Macedo, 2010	2	1	1	1	0	5
Martins, 2011	2	1	1	1	0	5
Mendoza Furtado, 2016	2	1	1	1	0	5
Merchan Villamizarto, 2020	1	1	0	1	2	5
Merchan, 2011	2	1	0	0	2	5
Miozzo, 2018	2	1	1	1	0	5
Neto, 2007	2	1	1	1	0	5
Neves, 2007	2	1	2	0	0	5
Nicolau, 2007	2	1	2	0	0	5
Nogueira, 2010	1	1	1	1	1	5
Oliveira, 2004	1	1	0	2	1	5
Pianta, 2015	2	1	0	1	1	5
Rao, 2017	2	1	2	0	0	5
Resnik, 2016	2	1	0	1	1	5
Rinaldi, 2013	2	1	2	0	0	5
Rueda-Clausen, 2009	2	1	1	0	1	5
Salvetti, 2008	2	1	2	0	0	5
Santana-Santos, 2014	2	1	1	1	0	5
Soares, 2009	2	1	2	0	0	5
Sperling, 2016	2	0	2	1	0	5
Arantes, 2020	1	1	0	1	1	4
Arazi H, 2010	1	1	2	0	0	4
Baraona, 2006	2	0	0	1	1	4
Barbosa JE, 2019	0	0	1	1	2	4
Blumel, 2003	2	0	0	1	1	4
Borges JC, 2010	1	1	0	2	0	4
Braga, 2015	1	1	1	0	1	4
Camargo, 2014	2	0	1	1	0	4

Caruso, 2016	1	0	1	1	1	4
Castanedo Amado, 2017	1	0	0	1	2	4
Castro Filho, 2015	1	0	2	1	0	4
Dutra da Silva, 2004	2	0	0	2	0	4
Fernandes, 2016	2	1	0	1	0	4
Ferreira, 2005	1	0	2	1	0	4
Ferreira, 2007	2	1	0	1	0	4
Franca, 2012	2	1	0	1	0	4
Garcia, 2012	2	0	0	2	0	4
Herdy, 2008	2	1	0	1	0	4
Hermes, 2015	2	1	0	1	0	4
Leite, 2010	2	0	0	1	1	4
Lopes, 2009	2	0	0	1	1	4
Luz, 2005	1	1	0	1	1	4
Mansur, 2001	2	1	0	1	0	4
Meirelles, 2006	2	0	0	1	1	4
Missel, 2009	2	0	0	1	1	4
Moreno, 2011	2	0	1	0	1	4
Navar, 2019	1	1	1	0	1	4
Oliveira, 2004	1	1	0	1	1	4
Peressoni, 2004	2	0	0	1	1	4
Prado, 2015	1	0	0	2	1	4
Rahmi, 2019	2	0	2	0	0	4
Santos, 2019	2	1	0	1	0	4
Serrano, 2010	2	0	2	0	0	4
Silva, 2012	1	0	2	1	0	4
Silva, 2014	1	1	0	2	0	4
Taty Zau , 2018	1	0	0	2	1	4
Theme-Filha, 2005	1	2	0	1	0	4
Vicario, 2005	1	1	0	1	1	4
Cruz, 2012	0	0	1	1	1	3
Da Silva, 2020	1	0	0	2	0	3
Dantas, 2002	1	1	0	1	0	3
Kampits, 2016	1	1	0	1	0	3
Kampits, 2016	1	0	0	1	1	3
Stein, 2004	1	0	0	1	1	3
Villalobos, 2013	1	0	0	1	1	3
Duque, 2016	1	1	0	0	0	2
Eibel, 2017	1	0	0	1	0	2
Gagliardi, 2016	1	0	0	1	0	2
Gama, 2010	1	0	0	1	0	2
Gama, 2012	1	0	0	1	0	2
Castro, 2004	0	0	0	0	1	1

Note: 0 points are attributed for high risk, 1 for moderate risk and 0 for high risk. Publications with a risk of bias score lower than 6 were not included in the review.

Supplementary File 3 C: Reasons for exclusion for articles with a risk of bias score <6.

Publication	Risk of bias score justification
Villalobos, 2013	Single centre, unclear inclusion and exclusion criteria.
Vicario, 2005	Pilot study, sample selected from small patient group.
Arazi , 2010	Unclear recruitment period and methodology data collection on medication use, single centre.
Arantes , 2020	Medication use was an exclusion criteria, single centre.
Theme-Filha, 2005	Participation rate field: lack on information on response or participation rate.
Barbosa, 2014	Single centre, unclear how data on diagnosis and medication was collected.
Taty Zau , 2018	Unclear origin of the study population, number of centres, and unclear methodology to collect medication use data.
Stein, 2004	Single centre, no detailed inclusion and exclusion criteria
Serrano, 2010	Unclear methodology to diagnose CAD is diagnosed and to measure medication use, unclear inclusion and exclusion criteria, single centre.
Santana-Santos, 2014	Single centre, unclear methodology to collect data, low participation rate,.
Salvetti, 2008	Unclear methodology to define diagnosis and to collect data on medication use, single centre.
Rinaldi, 2013	Unclear Inclusion and exclusion criteria and methodology to collect outcome data
Resnik, 2016	Unclear sampling method, limited information on participant's characteristics.
Rao, 2017	Unclear origin of the study population, vague inclusion and exclusion criteria, single centre, unclear methodology to collect data on medication use, unclear recruitment period.
Prado, 2015	Sample selection not at random, single centre.
Piantá, 2015	Single centre, unclear methodology to collect data on participants characteristics.
Pesaro, 2012	Another record (Pesaro 2012) provides data on the same study with larger sample size.
Pesaro , 2012	Cross-sectional design, single centre, limited information on how participant's characteristics and medication information was collected.
Peressoni, 2004	Unclear number of centres, unclear methodology to obtain baseline data.
Barbosa, 2019	Unclear methodology for data collection.
Blanco , 2016	Unclear methodology for data collection.
Blümel, 2003	Single centre, unclear sampling method.
Borges, 2010	Single centre, unclear how participants characteristics and medication data was obtained
Oliveira, 2004	Cross-sectional design, use of secondary data, single centre.

Nogueira, 2010	Single centre, retrospective design, sampling by convenience.
Nicolau, 2007	Unclear number of centres and origin of the study population, medication is part of the inclusion criteria, unclear method for data collection of participants characteristics and medication.
Neves, 2007	Single centre, unclear methodology to collect data on mediation and demographics, unknown study period.
Braga, 2015	Unclear data collection methodology on participant characteristics
Burdiat, 2016	Single centre
Camargo, 2014	Small subgroup in single centre
Castañeda-Amado, 2017	Retrospective design, unclear how participants characteristics data was collected, unclear sampling method.
Castello-Simões, 2015	Cross-sectional design, unknown study time or location, unclear how data on medication was obtained.
Castillo Costa, 2018	Unknown location, unclear how patient's characteristics data was obtained
Castro, 2004	Unknown study time or location, unclear how participants characteristics and medication information was collected.
Cardozo GG, 2015	Single centre, no sample size calculation
Caruso FR, 2016	Design: cross-sectional design
Cruz , 2009	Single centre and no sample size calculation
Cruz, 2012	Limited study design information
Dantas, 2002	Single centre, no sample size calculation
da Luz , 2005	Single centre, retrospective design, unclear patient selection method.
d a Silva , 2012	Retrospective study design, single centre, only patients with complete data included,
da Silva, 2014	Unclear follow-up time and data collection, single centre, no information on methodology to collect data on medication.
Da Silva, 2020	Cross-sectional design, single centre, unclear participation rate, limited methodology on how medication information is collected
Dayan , 2018	Retrospective data collection, single centre
de Mendonca Furtado, 2016	Single centre, unclear methodology to collect data on medication
de Oliveira, 2004	Retrospective study design, single centre, no participation rate information available
dos Santos, 2019	No participation rate information, single centre
Duque, 2016	Retrospective data collection, single centre, unclear recruitment time
Duque, 2018	Single centre, not representative sample (listed as limitation), optimal treatment was criteria for inclusion,
Dutra da Silva , 2004	Single centre, no inclusion or exclusion criteria, no PR information
Eibel , 2017	Single centre, no participation rate information, unclear recruitment time

Mansur, 2001	Single centre, unclear information on how participants characteristics information was obtained.
Macedo, 2010	-Single centre, no information on data collection methodology for patient characteristics and medication.
Meirelles, 2006	Single centre, no inclusion and exclusion criteria, high variability in study population, limited participation rate information, unclear methodology on how participants characteristics data is collected
Merchan, 2011	Single centre, limited information on participants characteristics.
Missel, 2009	Single centre, no inclusion/exclusion criteria. No information on how participants characteristics data is collected.
Moreno, 2011	Unknown study location, unclear how information on participants characteristics and medications obtained,
Navar, 2019	No information on data analysis, no information in number of centres, limited information on participants characteristics.
Neto, 2007	Single centre, unclear how participants characteristics data are obtained, limited participation rate information, unclear how medication data is collected.
Rueda-Clausen, 2009	No information on the number of centres, unknown study time and location.
Lopes, 2009	No indication on number of centres, unclear origin of the study population.
Lombo, 2010	Single centre, no inclusion and exclusion criteria.
Fantin, 2011	Single centres, no information on how outcome data is collected.
Feguri , 2012	Single centre, no information on participation rate, no information on how outcome data is collected.
Fernandes , 2006	Single centre, no participation rate information, limited information on how medication information is collected.
Fernandes , 2008	Single centre, no information on how participants' characteristics or medication data were collected.
Fernandes , 2016	Single centre, unclear participation rate information, no information on how medication data is collected.
Ferreira , 2005	Unclear study design
Ferreira , 2007	Single centre, no participation rate information, unclear how data on participant's characteristics is collected
Leite, 2010	Single centre, vague inclusion and exclusion criteria, unknown study period.
Ferreira, 2017	Retrospective design, single centre, unclear how medication data is collected
França , 2012	Single centre, unclear recruitment period, no information on how medication data is collected
Gagliardi, 2016	Single centre and narrow target group.
Gama , 2010	Cross-sectional data, single centre, no participation rate information.
Gama , 2012	Cross-sectional design, single centre
Garcia , 2012	Single centre, unclear method to select patient, unclear how patients characteristics and medication information was obtained.
Rahmi, 2019	Single centre, unknown study time or location, no information on how medication data is collected.
Baraona, 2006	Single centre, no information on participation rate.

Garlet, 2017	Retrospective design, single centre
Herdy , 2008	Single centre
Hermes , 2015	Single centre, unclear sampling method, no recruitment time reported.
Kampits , 2016	Retrospective design, single centre.
Kampits , 2016	Cross-sectional design, data collection from medical records, single centre.
Merchán Villamizarto , 2020	Cross-sectional design, non-random sampling, no information on participation rate
